# Differential burst dynamics of slow and fast gamma rhythms in macaque primary visual cortex

**DOI:** 10.1162/IMAG.a.1404

**Published:** 2026-10-22

**Authors:** Vignesh Raju, Supratim Ray

**Affiliations:** Centre for Neuroscience, Indian Institute of Science, Bengaluru, India

**Keywords:** gamma, oscillations, bursts, LFP, Wilson–Cowan

## Abstract

Gamma oscillations have been ubiquitously observed across a wide spectrum of brain areas in multiple species. They tend to occur intermittently in the form of bursts, rather than being produced as sustained and continuous rhythmic activity. Recent studies have shown that large visual sinusoidal gratings elicit two distinct gamma rhythms, namely, slow (≈20–35 Hz) and fast gamma (≈40–65 Hz), in the primary visual cortex (V1) of non-human primates. However, their mechanisms of generation and potential functional role in cortical processing remain unclear. Details of their burst signatures could potentially provide crucial insights about how the two rhythms influence network dynamics. Therefore, we computed burst statistics (durations and latencies) of simultaneously induced slow and fast gamma rhythms in the local field potential (LFP) recorded from area V1 of two adult female bonnet monkeys using several burst estimation methods. We found that slow gamma rhythm exhibited significantly longer burst durations and longer latencies than fast gamma rhythm. Slow gamma also exhibited higher long-range synchrony than fast gamma, as estimated by coherence and weighted phase lag index (WPLI). Interestingly, longer burst length of slow gamma could be replicated in a recently developed noisy Wilson–Cowan network model by simply changing the firing-rate time constant of the corresponding inhibitory interneuronal population, which led to both slower and longer bursts. These results are consistent with the hypothesis that the two oscillations are generated by different inter-neuronal classes that operate over different temporal and spatial scales of integration.

## 1 Introduction

Gamma rhythms (typically, 30–70 Hz) have been associated with diverse cognitive phenomena, including memory (Lisman & Idiart, 1995), selective attention (Jensen et al., 2007), and feature binding through synchronization (Han et al., 2022; Singer & Gray, 1995). Cognitive impairments such as Alzheimer’s disease (AD), schizophrenia, and attention-deficit hyperactivity disorder (ADHD) have been correlated with atypical gamma oscillations (Herrmann & Demiralp, 2005; Murty et al., 2021; Uhlhaas & Singer, 2010). Notably, a decline in the power of gamma oscillations, even with normal aging in healthy individuals, has been recently reported (Murty et al., 2020). They are widely believed to be generated due to network interactions between inhibitory and excitatory populations of neurons.

Experimental studies have revealed the presence of multiple gamma rhythms in the rodent hippocampus (Belluscio et al., 2012; Colgin et al., 2009) and primary visual cortex (V1) (Chen et al., 2017; Meyerolbersleben et al., 2025; Niell & Stryker, 2010; Perrenoud et al., 2025; Saleem et al., 2017; Veit et al., 2017). These have been associated with distinct processes, including thalamic activation as well as interaction with different classes of inhibitory interneurons (see Section 4.1 for a detailed discussion). In non-human primate V1, large visual gratings have been shown to induce two distinct gamma rhythms: slow (≈20–35 Hz) and fast gamma (≈40–65 Hz) (Murty et al., 2018), although there are important differences between primate and rodent gamma (as discussed later). Furthermore, these distinct gamma rhythms are tuned to stimulus features such as spatial frequency and contrast (Han, Wang, Yang, et al., 2021).

A separate line of investigation has revealed gamma oscillations to be generated stochastically, in the form of bursts in macaque V1 (Chandran KS et al., 2018; Xing et al., 2012). Such brief bursts are observed in multiple bands (such as beta and gamma) and in multiple brain regions, and have been hypothesized to play a role in encoding and retrieval during the working memory paradigm in the prefrontal cortex of monkeys (Lundqvist et al., 2016) and in facilitating information flow across cortical areas on a cycle-by-cycle basis (Palmigiano et al., 2017). In rodent V1, such gamma bursts have also been linked to distinct features of visual stimuli (Meyerolbersleben et al., 2025) and behavioral context (Perrenoud et al., 2025), although whether gamma oscillations play a causal role in information processing or reflect ongoing excitation–inhibition interactions in cortex is still a matter of debate (Cho et al., 2020; Ray & Maunsell, 2010, 2015). Such bursty oscillations have been modeled using Wilson–Cowan (WC) models that are driven by Poisson inputs (Jia et al., 2013) or a non-linear sustained limit cycle model (Jadi & Sejnowski, 2014) with Ornstein–Uhlenbeck (OU) noise inputs (Krishnakumaran & Ray, 2023). Further, recent models have shown that the burst profiles could be critically influenced by the underlying network connectivity parameters and the appropriate regime of oscillations (Powanwe & Longtin, 2019). Hence, a detailed characterization of burst statistics could convey important information about the underlying neural circuitry.

Here, we characterized the burst statistics, such as duration and temporal distribution, of simultaneously induced slow and fast gamma bursts. We recorded local field potential (LFP) using microelectrodes from the area V1 of two awake adult female bonnet monkeys performing a passive fixation task, while they viewed large-sized sinusoidal gratings that induced both the gamma rhythms. We estimated burst lengths of slow and fast gamma rhythms present in the dataset using the Matching Pursuit (MP) algorithm, an iterative and greedy signal decomposition technique that has been shown to robustly compute burst durations (Chandran KS et al., 2018). We also used other methods, including traditional methods such as Hilbert Transform, as well as a recently developed compressed sensing technique that can detect bursts using smaller but dynamic dictionaries (Anand et al., 2025). Further, we studied the extent of synchronization in slow and fast gamma by analyzing phase coherence and weighted-phase lag index (WPLI) profiles. Finally, we tested whether the burst characteristics could be explained in the OU noise model (Krishnakumaran & Ray, 2023) by varying the firing rate time constant of the inhibitory neuronal population.

## 2 Materials and Methods

### 2.1 Animal preparation and recording

Two adult female bonnet monkeys (*Macaca radiata*) weighing 4 and 3.3 kg were used in this study. Experimental protocols used for the animals were in accordance with the guidelines approved by the Committee for the Control and Supervision of Experiments on Animals (CCSEA) and the Institutional Animal Ethics Committee (IAEC) of the Indian Institute of Science (IISc). A head post made of titanium was surgically implanted in both monkeys. It was then followed by a period of training for a visual fixation task. Post-training, a hybrid array of microelectrodes (Utah array), which consisted of 81 microelectrodes (9 x 9) and 9 ECoG (3 x 3) electrodes, was implanted in the primary visual cortex (V1) of the right cerebral hemisphere under anesthesia. Microelectrodes (length = 1 mm) had an interelectrode distance of 400 µm. Grid placement was approximately set at ≈15 mm lateral from midline in V1 and ≈15 mm rostral from the occipital ridge. The placement of reference wires was over the dura near the recording location, or they were wrapped around titanium screws on the surface of the skull near the craniotomy.

To get LFP data, the raw data obtained were band-pass filtered between 0.3 Hz (Butterworth filter, first order, analog) and 500 Hz (Butterworth filter, fourth order, digital), digitized at 16-bit resolution (sampled at 2 kHz). Further, LFP signals were decimated by a factor of 8 (sampling frequency became 250 Hz) using the MATLAB function “resample” to reduce the runtime of the burst detection algorithms.

### 2.2 Behavioral task and experimental design

The monkeys sat on a chair inside a Faraday cage enclosure to shield them from external electrical noise, and their heads were fixed by the head post while performing the visual fixation task. Monkeys were required to fixate their gaze within 2^o^ of a small central dot (0.05^o^ or 0.1^o^ diameter) displayed on an LCD monitor screen (BenQ XL2411, LCD, 1280 X 720 pixels, 100 Hz refresh rate, gamma corrected). The monitor was placed 50 cm away from the eyes of the subjects. A single trial block consisted of an initial blank period of 1000 ms, followed by a display of a series of two to three stimuli in succession for 800 ms each, with an interstimulus interval of 700 ms. On successful fixation throughout the block, they were given a juice reward at the end.

Full-screen static gratings (covering ≈56^o^ (width) and 33^o^ (height) of the visual field) were the stimuli presented at full contrast, one of the eight orientations (0^o^, 22.5^o^, 45^o^, 67.5^o^, 90^o^, 112.5^o^, 135^o^, and 157.5^o^) and one of the five spatial frequencies (0.5, 1, 2, 4, and 8 cycles per degree). They were presented in pseudorandom order. The average number of trials for each pair of spatial frequency and orientation was 33 (28–36) for Monkey 1 and 42 (37–45) for Monkey 2. This dataset has been used in our previous studies (Dubey & Ray, 2020; Gautham & Ray, 2024; Prabhu & Ray, 2024). The receptive fields (RFs) of the neurons contributing to signals of microelectrodes were centered in the lower left quadrant of the visual field at an eccentricity of ~3° to ~4.5° in Monkey 1 and ~1.4° to ~1.75° in Monkey 2.

### 2.3 Electrode selection

We limited our further analysis to electrodes, whose RF estimates were steady over the period of many days (SD less than 0.1^o^), similar to our earlier studies (Dubey & Ray, 2020; Murty et al., 2018). This resulted in 77 and 31 electrodes for Monkey 1 and Monkey 2, respectively.

### 2.4 Data analysis

Our entire data analysis was performed using custom codes written in MATLAB R2024b (MathWorks, RRID: SCR_001622). For the construction of the time–frequency power spectrogram, we used a moving window of length 250 ms and a step size of 25 ms, leading to a frequency resolution of 4 Hz. For the power spectral densities (PSDs), the baseline period was defined as -500 to 0 ms of stimulus onset, and the stimulus period was considered to be 250 to 750 ms for each stimulus presentation to avoid stimulus-onset related transients in the first 250 ms. PSDs were computed for the stimulus and baseline periods using the multitaper method (single taper) using the Chronux toolbox (Mitra & Bokil, 2008) (http://chronux.org/, RRID: SCR_005547). The frequency resolution for estimated PSDs was 2 Hz.

The full-screen stimuli induced slow and fast gamma waves that depended on the orientation and spatial frequency of the stimuli. We restricted our entire analysis to stimuli with a spatial frequency of 1 CPD, as both slow and fast gamma were induced across different stimulus orientations (Supplementary Fig. S1B, D). Based on the average PSD traces, the slow gamma frequency range was considered to be between 20 and 32 Hz for Monkey 1 and 20 and 38 Hz for Monkey 2 (Supplementary Fig. S1A, C). Similarly, the fast gamma frequency range was considered between 36 and 65 Hz for Monkey 1 and 42 and 65 Hz for Monkey 2. Note that the presence of two bumps in the trial-averaged PSDs does not mean that slow and fast gamma bursts co-occur in individual trials. The power tuning curves of fast and slow gamma bands were constructed by averaging the power in these bands, as done in previous studies (Murty et al., 2018).

### 2.5 Generation of synthetic LFP signal

For testing the performance of various burst detection methods, we generated synthetic LFP signals by injecting Gabor atoms (Gaussian-modulated sinusoidal waveforms, mimicking gamma bursts) of pre-determined fixed durations into the spontaneous LFP that was recorded when no stimulus was shown, similar to previous studies (Anand et al., 2025; Chandran KS et al., 2018). In these previous studies, we had a condition where a stimulus of 0% contrast (i.e., no stimulus) was presented, which was used to get long segments of “spontaneous” LFP. Since such a condition was not present in the current dataset, we simulated long spontaneous LFP segments (same as the total trial duration in the actual dataset) by interpolating the Fast Fourier Transform (FFT) of the baseline segment (500 ms duration). Specifically, to generate spontaneous LFP segments of longer durations, we interpolated the baseline LFP FFT amplitudes using the MATLAB function “interp1”, which were further normalized appropriately to keep the total energy fixed. Phase angles corresponding to the interpolated points were randomly generated. Next, we computed the inverse Fourier transform to generate the full-length spontaneous LFP segment in the time domain. Four times the standard deviation of an injected Gabor atom (covering around 95% of the energy of the atom) were considered as the length of the burst. The center frequency of injected bursts was chosen from a uniform distribution between 20 and 65 Hz, as both the slow and fast gamma “bumps” were primarily localized in the defined range for both Monkeys. While injecting longer bursts (>250 ms), the number of bursts per trial was fixed to one. For bursts shorter than 250 ms, the number of bursts injected per trial was estimated from a Poisson distribution, with its expected value equal to the ratio of length of stimulus period (500 ms: 0.25–0.75 seconds) and length of bursts to be injected. The selection of the amplitude, time center, and other metrics of the injected synthetic burst was made using the same criteria as our previous study (Chandran KS et al., 2018). Bursts of fixed durations ranging from 50 ms to 400 ms (with 50-ms step size) were injected. The average frequency spectrum of the synthetic LFP trials (separately for each burst length injected) was appropriately scaled with a constant to match the average PSD of the actual dataset, in the frequency range of 20–65 Hz. For the evaluation of performance of the three methods on the synthetic dataset, we computed F-1 score, which is defined as the harmonic mean of recall (sensitivity) and precision. A detected burst was considered as a true positive, if its duration was within ±40 ms of the actual duration of the injected burst.

### 2.6 Burst detection methods

#### 2.6.1 Matching pursuit algorithm (MP)

Matching pursuit (Chandran KS et al., 2016, 2018; Durka et al., 2001; Mallat & Zhifeng Zhang, 1993) is an iterative signal decomposition-based algorithm. Adopting a greedy approach, it approximates a signal in the time domain as a linear combination of waveforms called atoms. Initially, an extensive and overcomplete dictionary of atoms (Gabor, Fourier, and delta) is first made by scaling, shifting, and modulating a single mother atom:

$$g_{\gamma }\left(t\right)=\frac{1}{\sqrt{a}}g\left(\frac{t-u}{a}\right)e^{j\epsilon t}.$$

Here, $g_{\gamma }\left(t\right)$ is an element (waveform or atom) of the dictionary (D), which we obtain by modulating the single mother atom g(t) by γ=(u,a,ϵ) that corresponds to the given values of parameters: translation (u), scale (a) , and modulation (ϵ). Normalization factor $\left(\frac{1}{\sqrt{\left(\text{a}\right)}}\right)$ ensures that the energy of the atom is unity.

$$g_{\gamma m}=arg\;max_{g_{\gamma i}\in D}\left|\langle R_{m},g_{\gamma i}\rangle \right|$$

$$R_{n}=\langle R_{n},g_{\gamma n}\rangle g_{\gamma n}+R_{n+1}.$$

Here, f(t) is the signal, while $R_{m}\text{ and}\;g_{\gamma m}$ are the residue and selected atom at the m^th^ iteration, respectively. At the initial iteration, the residue is set equal to the input signal itself $\left(R_{0}=f\right)$. This is followed by the computation of the inner product between the residue and all the atoms in the dictionary. The atom that has the largest inner product is selected. For subsequent iterations, the residue is set equal to the difference between the previous residue and the inner product between the chosen atom and the previous residue multiplied by the atom itself (approximation or projection). This procedure is repeated over a defined number of iterations. Upon completion of n iterations, the signal is decomposed as follows:

$$f=\sum_{m=0}^{n-1}\langle R_{m},g_{\gamma m}\rangle g_{\gamma m}+R_{n}.$$

We used matching pursuit with a stochastic dictionary, in which each of the scale, shift, and modulation parameters is sampled uniformly. Therefore, atoms of all durations are present at both low and high frequencies. For our entire analysis, the dictionary size was 2.5 million, which was sufficient to sample the parametric space effectively (Anand et al., 2025; Chandran KS et al., 2018). We used 120 iterations for the MP algorithm and achieved reasonable convergence (in terms of energy of the signal) with the given number of iterations. Prior to MP decomposition, the input LFP signal was band-pass filtered (using MATLAB function “filtfilt”) between 15 and 80 Hz to decrease the required number of iterations and, therefore, to optimize the runtime of the algorithm. The scale parameter of a burst was used to compute its duration, which was taken as four times the standard deviation (proportional to the scale parameter) of the Gabor atom (covering approximately 95% of its energy); choosing a different number would increase or decrease the burst lengths uniformly but otherwise not affect the comparison between burst lengths. Relevant bursts were selected according to their frequency center (within our frequency range of interest) and time center (within the stimulus period). Further, we filtered estimated bursts based on a power threshold criterion (relative to baseline) similar to one used in Chandran KS et al. (2018). This power threshold was specifically computed for the slow and fast gamma frequency ranges. We have previously shown that MP gives accurate results for a wide range of threshold values, unlike more traditional methods (see figure 4 of Chandran KS et al. (2018)).

#### 2.6.2 Hilbert transform (HT)

For burst detection using the Hilbert transform (Lundqvist wet al., 2016), the instantaneous power was considered as the square of the magnitude of the analytic signal, given as follows:

$$A_{f}\left(t\right)=f\left(t\right)+jH\left(f\left(t\right)\right).$$

Here, f(t) is defined as the band-pass filtered signal (in the frequency range of interest), using MATLAB function “filtfilt” (Butterworth filter-order 4), and H is the Hilbert transform. The instantaneous power was taken as the square of the instantaneous amplitude. Occurrences of gamma bursts (seed points) were located when the power exceeded three times the median power of the baseline signal (mBL). The start and end points of each burst were defined at the points where the power went below 1.5 times the mBL from that seed point, on either side of the seed point (similar metric as used in Feingold et al. (2015) for beta burst detection).

#### 2.6.3 Orthogonal matching pursuit-Gabor expansion with atomic reassignment (OMP-GEAR)

OMP-GEAR is also an iterative algorithm, where it directly fits the residue (initially, the signal itself) to a new atom, using the magnitude of the inner product between the residue and the initial atom found by the canonical OMP method. It uses a unique parameter reassignment method for further refining the selected atom, thus not limiting the search space to only a finite-sized dictionary (for more details, see Anand et al., 2025). The dictionary size used for OMP-GEAR was 1.5 million, although smaller dictionaries also provide accurate results (Anand et al., 2025). Other specifics, such as the number of iterations (120), threshold criterion, and broad frequency range (15 and 80 Hz) of initial band-pass filtering, were the same as those used for the MP algorithm.

Rare occurrences of spurious bursts longer than 800 ms (stimulus duration) were excluded from the analysis across all methods. As shown in Figure 2 and Supplementary Figure S4, less than 5% of the bursts were longer than 800 ms. We have previously shown stimuli of longer durations (2 seconds) and found that the burst distribution remains similar to the one obtained here (see figure 7B of Chandran KS et al. (2018) for burst distribution for 2-second long stimuli).

### 2.7 Analysis of burst dynamics and statistical tests

For comparison of slow and fast gamma burst durations, we considered only those electrodes (for a given stimulus condition) for which more than 20 bursts were detected for both rhythms. Most of the electrodes that were previously selected based on receptive field estimation fulfilled the above criteria for both monkeys across all the methods.

The burst length distributions of slow and fast gamma were compared with a two-sample Kolmogorov–Smirnov (KS) test. The median durations and mean onset times (both electrode-wise) of slow and fast gamma bursts were analyzed using the one-tailed two-sample Wilcoxon signed-rank test and paired t-test, respectively. Throughout our study, the standard error around the median was computed using the standard bootstrap method, with 1000 iterations.

The power matching for slow and fast gamma frequency ranges across the electrodes was performed using a histogram bin-height matching method (bin width: 0.5 dB (power)), as done in a previous study (Kumar & Ray, 2023). For each bin, we randomly selected electrodes (using MATLAB function “randsample”) from the rhythm having a longer bin height to make the bin height of both distributions equal. This resulted in two equal-sized groups of electrodes with similar fast and slow gamma power. Burst lengths of slow and fast gamma for the set of power-matched electrodes were compared using the one-tailed two-sample Wilcoxon rank-sum test across all methods. We computed the number of cycles contained in a burst as the burst duration multiplied by its center frequency. Further, burst occupancy for a trial was estimated as the total (union) time occupied by bursts.

### 2.8 Connectivity estimation

We computed the phase locking value (PLV) or phase coherence (Lachaux et al., 1999), and the weighted phase lag index (WPLI) (Vinck et al., 2011) using the Fieldtrip Toolbox (Oostenveld et al., 2011; RRID: SCR_004849). The PLV estimate between two signals from a pair of electrodes is defined as follows:

$$PLV_{f}=\frac{1}{N}\left|\sum_{t=1}^{N}e^{j\left(\theta_{t}\left(f\right)-\phi_{t}\left(f\right)\right)}\right|.$$

Here, N denotes the total number of trials (in case of using a single taper), $\theta_{t}\left(f\right)$ and $\phi_{t}\left(f\right)$ are the phase angles of the estimated spectrum of the two signals, corresponding to f (frequency) and t (trial number). Further, we employed the WPLI metric in order to infer about the spatial scales of origin of the rhythm, as it has been shown to be a robust indicator of synchrony against noise and volume conduction (Vinck et al., 2011), and is computed as follows:

$$WPLI_{f}=\frac{\left|E\left\{Im\left(X_{f}\right)\right\}\right|}{E\left\{\left|Im\left(X_{f}\right)\right|\right\}}.$$

Here, $Im\left(X_{f}\right)$ represents the imaginary component of the cross-spectrum between the two signals corresponding to f (frequency), and E denotes the expected value or mean across trials. These connectivity measures were estimated for each pair of electrodes in the dataset. These pairs were further classified according to interelectrode distances. The baseline and stimulus periods were taken the same as above, resulting in a frequency resolution of 2 Hz. The number of tapers used for PLV and WPLI computation was 1 and 3, respectively.

### 2.9 Model implementation details

We used a model implemented recently in our previous studies (Krishnakumaran & Ray, 2023; Krishnakumaran et al., 2025). It is an extension of the Jadi and Sejnowski (JS) model (Jadi & Sejnowski, 2014), which is a basic firing rate model consisting of an inhibitory and an excitatory population of neurons with a sigmoidal activation function, which, for a proper choice of inputs, operates as an Inhibition Stabilized Network (ISN). The model was able to generate gamma oscillations that replicate the decrease in peak frequency and increase in power with increasing size of the stimulus, as reported by previous studies in V1 (Gieselmann & Thiele, 2008), as well as an increase in the peak frequency with contrast (Ray & Maunsell, 2010). The population firing rates $r_{E}$ and $r_{I}$ of excitatory and inhibitory neurons, respectively, evolve according to the following differential equations in the model:

$$\tau_{E}\frac{dr_{E}}{dt}=-r_{E}+f_{E}\left(W_{EE}r_{E}-W_{EI}r_{I}+i_{E}\right)$$

$$\tau_{I}\frac{dr_{I}}{dt}=-r_{I}+f_{I}\left(W_{IE}r_{E}-W_{II}r_{I}+i_{I}\right)$$

$$f_{P}\left(x\right)=\frac{1}{1+e^{m_{P}.\left(c_{P}-x\right)}}-\frac{1}{1+e^{m_{P}.c_{P}}}.$$

Here, the subscript P refers to the specific population, which could be either E (excitatory) or I (inhibitory). The original JS model produced sustained continuous limit cycles of gamma oscillations, as it employed constant input drives. Recruiting noisy input drives (specifically, Ornstein–Uhlenbeck (OU) noise) in the JS model has been shown to generate bursty gamma rhythm, with burst statistics similar to those observed in vivo (Krishnakumaran & Ray, 2023). The OU noise formulation of input drives $i_{E}$ and $i_{I}$ is given as follows:

$$\frac{1}{2\text{π}\theta_{E}}\frac{di_{E}}{dt}=-i_{E}+I_{E}+\sigma_{E\;}N\left(0,1\right)$$

$$\frac{1}{2\text{π}\theta_{I}}\frac{di_{I}}{dt}=-i_{I}+I_{I}+\sigma_{I\;}N\left(0,1\right).$$

Here, $I_{E}$ and $I_{I}$ refer to the steady-state mean values of $i_{E}$ and $i_{I},$ respectively, while N(0,1) denotes that at each time point, a value is generated from the standard Gaussian distribution. This formulation is essentially equivalent to additive white noise passed through a first-order low-pass filter, which has a cutoff frequency at $\theta_{P}$ corresponding to the population P.

We independently generated slow and fast gamma by suitably varying $\tau_{I}$, that is, the firing rate time constant of inhibitory neuronal population, which determines the center frequency of the generated oscillation. For slow gamma, $\tau_{I}$ was taken equal to $\tau_{SOM}\approx 15.4$ ms, while for fast gamma, $\tau_{I}$ was set as $\;\tau_{PV}\approx 7.7$ ms, where SOM and PV refer to somatostatin and parvalbumin-positive interneurons, respectively, which have been shown to play a role in specifically generating the two rhythms. These values of the firing rate time constant of the specific interneurons are similar to those used in previous studies (Garcia Del Molino et al., 2017; Krishnakumaran & Ray, 2023). Furthermore, $W_{II}$ for slow gamma simulation was set to zero, as there is generally no recurrent connection among SOM interneurons in V1 (Bos et al., 2025; Garcia Del Molino et al., 2017; Pfeffer et al., 2013). The remaining parameters were kept the same across the two simulations and were the same as used in our previous study (Tables 1 and 2; Krishnakumaran & Ray, 2023).

**Table 1.** Parameter values used in the model for generating fast gamma.

W_EE_	16
W_EI_	26
W_IE_	20
**W_II_**	1
m_E_	1
m_I_	1
c_E_	7.5
c_I_	18
$\tau_{E}$ (ms)	15.4
$\tau_{I}=\tau_{PV}\;$ (ms)	7.7

**Table 2.** Parameter values used in the model for generating slow gamma.

W_EE_	16
W_EI_	26
W_IE_	20
**W_II_**	0
m_E_	1
m_I_	1
c_E_	7.5
c_I_	18
$\tau_{E}$ (ms)	15.4
$\tau_{I}=\tau_{SOM}\;$ (ms)	15.4

We simulated the model with 128 distinct parameter combinations, obtained by varying the input drive ($I_{E}=I_{I}\in \left\{7,\;8,\;9,\;10,\;11,\;12\right\})$ and independently varying $\theta_{P}$ ($\theta_{E}$, $\theta_{I}$ ∈{1, 4, 16, 64}) . The standard deviation of the noise (σ_P_) was set equal to the steady-state mean input $I_{P}$. The range of parameters selected was similar to that in previous studies (Jadi & Sejnowski, 2014; Krishnakumaran & Ray, 2023) to ensure that the slow and fast gamma were robustly generated within physiologically observed frequency ranges in V1. Each parametric combination was simulated for 50 iterations for both slow and fast gamma, using the forward Euler method (time step size: 2 x 10^-5^ seconds). Each iteration had a timespan from -2.047 to 2.048 seconds, where the input drives and variances were set to zero from -2.047 to 0 seconds, then instantaneously set to the desired non-zero value from 0 to 1.5 seconds, following which they were again set to zero. This is equivalent to a stimulus presentation duration of 1.5 seconds. We considered $-r_{E}-\;r_{I}$ as the proxy for LFP, similar to our previous studies (Krishnakumaran & Ray, 2023; Krishnakumaran et al., 2022). Further, it was passed through a fourth-order low pass Butterworth filter (cutoff frequency at 200 Hz) and downsampled by a factor of 200, resulting in a sampling frequency of 250 Hz (same as that in our actual dataset). Time–frequency spectra analysis and PSD estimation of the simulated model output were performed similarly to that described above for the actual LFP. Simulated slow gamma LFP proxy was band-pass filtered (using MATLAB function “filtfilt”) in the frequency range between 10 and 50 Hz, followed by MP analysis using 50 iterations. Fast gamma LFP proxy was filtered between 30 and 90 Hz, followed by MP analysis using 100 iterations. The number of iterations was chosen to ensure that the MP algorithm achieves optimal convergence in decomposing the energy of the signal (band-pass filtered in the frequency range of interest).

Stimulus time period was defined as 0.25 to 1.25 seconds (to avoid stimulus-onset related transients). The slow gamma frequency range was considered to be between 20 and 40 Hz, and the fast gamma frequency range was considered between 40 and 80 Hz. As mentioned above, relevant bursts were selected according to their frequency center (within our frequency range of interest), time center (within the stimulus period), and a power-based threshold. Any spurious bursts much longer than the stimulus duration were excluded from the analysis. For the model, due to the absence of a non-zero baseline power, the normalized power of the specific gamma band was estimated by dividing the maximum power (peak) in the frequency band by the average power of both ends of the frequency band (based on PSD), similar to a recent modeling study (Han, Wang, Wu, et al., 2021). Subsequently, power matching and other statistical analyses were performed across different parametric conditions in the same way as described above for the actual dataset.

## 3 Results

### 3.1 Generation of slow and fast gamma rhythms by large visual stimuli

We analyzed LFP signals recorded from area V1 of two female monkeys while they viewed full-screen sinusoidal gratings of varying orientations and spatial frequencies. As shown previously, these full-field stimuli induced two distinct gamma rhythms, with distinct tuning preferences for both orientation (Supplementary Fig. S1) and spatial frequency (Dubey & Ray, 2020; Murty et al., 2018).

### 3.2 Robust burst length estimation by MP

We evaluated the performance of three methods (HT, MP, and OMP-GEAR) for burst detection on the synthetic LFP signal, constructed by injecting Gabor atoms into spontaneous LFP (see methods for details). We have done a detailed comparison of various burst detection algorithms in our previous studies (Anand et al., 2025; Chandran KS et al., 2018) and here discuss the same in Supplementary Discussion and Figure S2.

We proceeded with the MP algorithm to estimate burst durations in our dataset. Figure 1A shows the broadband LFP trace from a single trial (black trace) overlaid with its band-pass filtered versions in the slow (blue trace) and fast (orange) gamma frequency range. The corresponding time–frequency (TF) spectrum (based on MP decomposition of the signal filtered between 15 and 80 Hz) is shown in Figure 1B. The gamma bursts identified by the MP algorithm in the slow and fast gamma ranges are shown as solid line segments in Figure 1A and B. In this trial, we observed two short bursts of fast gamma and a single long delayed slow gamma burst, with minimal overlap.

**Fig. 1.**
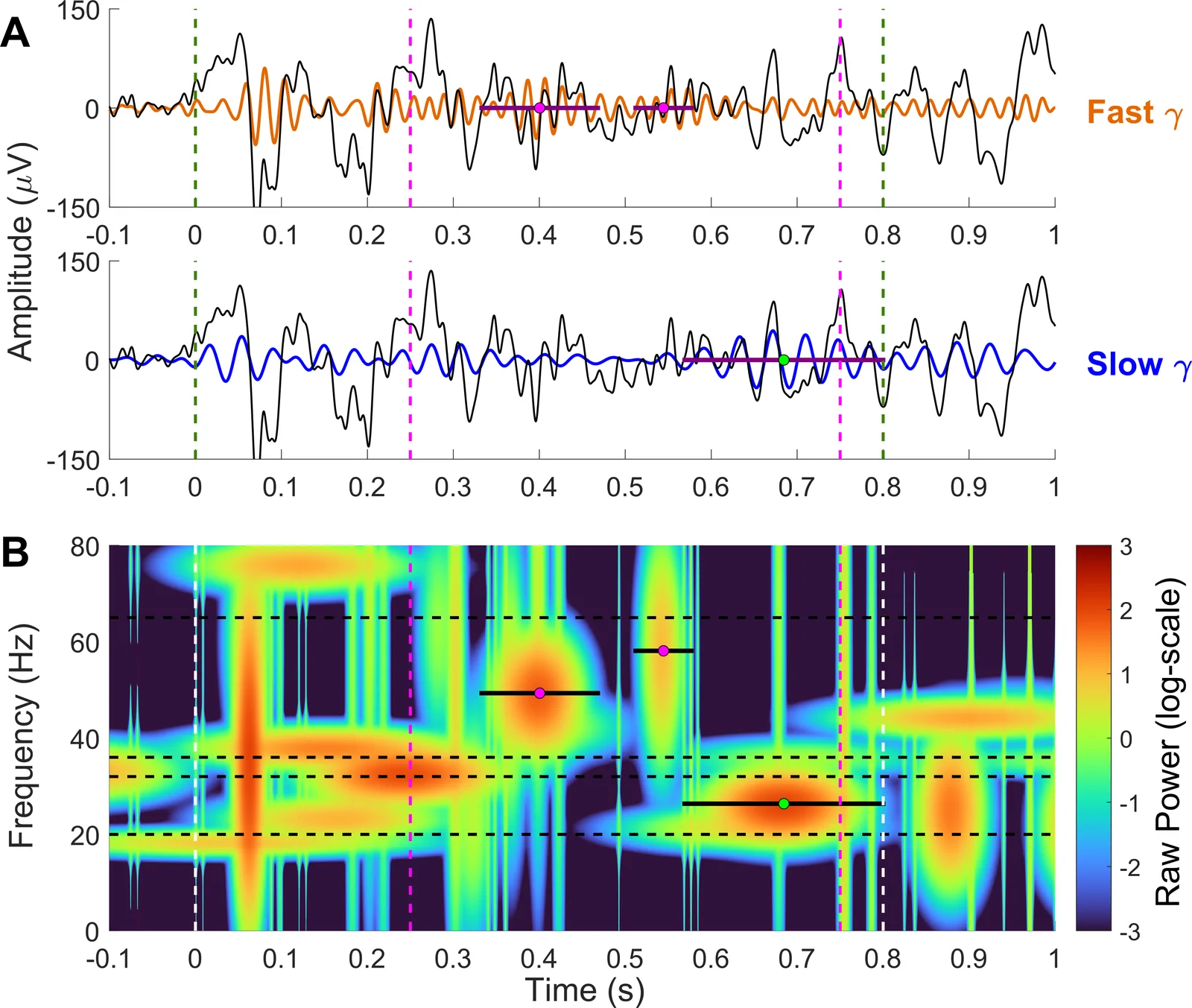
Gamma burst estimation by MP. (A) The black trace in both panels represents the broadband LFP trace (filtered between 3 and 100 Hz) of a single trial, from an example electrode, overlaid with the same signal band-pass filtered in fast (orange; top panel) and slow gamma (blue; bottom panel) ranges, respectively. The violet solid lines represent the burst durations detected by MP for this single trial (colored dot is the time center of the burst). (B) The time–frequency (TF) spectrum of the same single trial (with the signal filtered between 15 and 80 Hz) estimated by MP. The black line segments represent the bursts detected by MP for this single trial. Vertical magenta dotted lines represent the considered stimulus period (0.25–0.75 seconds), while the vertical green (A) or white (B) dotted lines indicate the stimulus onset (0 seconds) and offset (0.8 seconds). Horizontal black dotted lines represent the frequency ranges of slow (20–32 Hz) and fast gamma (36–65 Hz) for Monkey 1. Colorbar indicates the estimated power on a logarithmic scale.

Supplementary Figure S3A shows another trial in which two short fast gamma bursts and one long slow gamma burst were detected, but with substantial overlap, emphasizing the trial-to-trial variability present in gamma burst dynamics. The signal was initially band-pass filtered between 15 and 80 Hz prior to MP-based decomposition to decrease the run time of MP, as fewer iterations are needed in this case (see Methods section for details). However, such filtering can also generate spurious oscillations that may not be present in the original signal. To rule out this possibility, we also computed the TF plot of the raw (unfiltered) signal with a higher number of iterations, and found that the detected bursts remained consistent (Supplementary Fig. S3B), similar to a previous study (Chandran KS et al., 2018). We also constructed the TF plot using conventional multi-taper with a long window (Supplementary Fig. S3C; *N* = 1 taper, window length of 200 ms, window step size of 20 ms), which also showed more power at the times where bursts were detected by the MP algorithm. Such long windows smooth the spectrum and, therefore, cannot be used to detect short bursts in the data. Previously, TF spectra have been constructed with very short window lengths for burst detection (e.g., Continuous Gabor Transform with a window width of 12.5 ms; Supplementary Fig. S3D; Xing et al., 2012). This resulted in a very noisy TF spectrum due to the inherent variability in spectral estimation (Chandran KS et al., 2018). On increasing the window width to 25 ms, patches of high power appeared around the MP-detected bursts (Supplementary Fig. S3E). Note that MP, being an overcomplete signal decomposition method, does not have a fixed window size.

### 3.3 Longer burst lengths are observed for slow gamma rhythm

Since both slow and fast gamma power depended on stimulus orientation (Supplementary Fig. S1), we first compared the burst durations for an orientation of 157.5^o^, for which power was comparable in the two gamma bands for Monkey 1 (for Monkey 2, slow gamma was weaker than fast gamma at all orientations). The histogram of burst lengths combined across all electrodes for both the rhythms (Fig. 2A, E) showed a characteristic peak (mode) at short burst durations, along with a right tail, consistent with previous studies where a single gamma rhythm was analyzed (Chandran KS et al., 2018; Krishnakumaran & Ray, 2023).

**Fig. 2.**
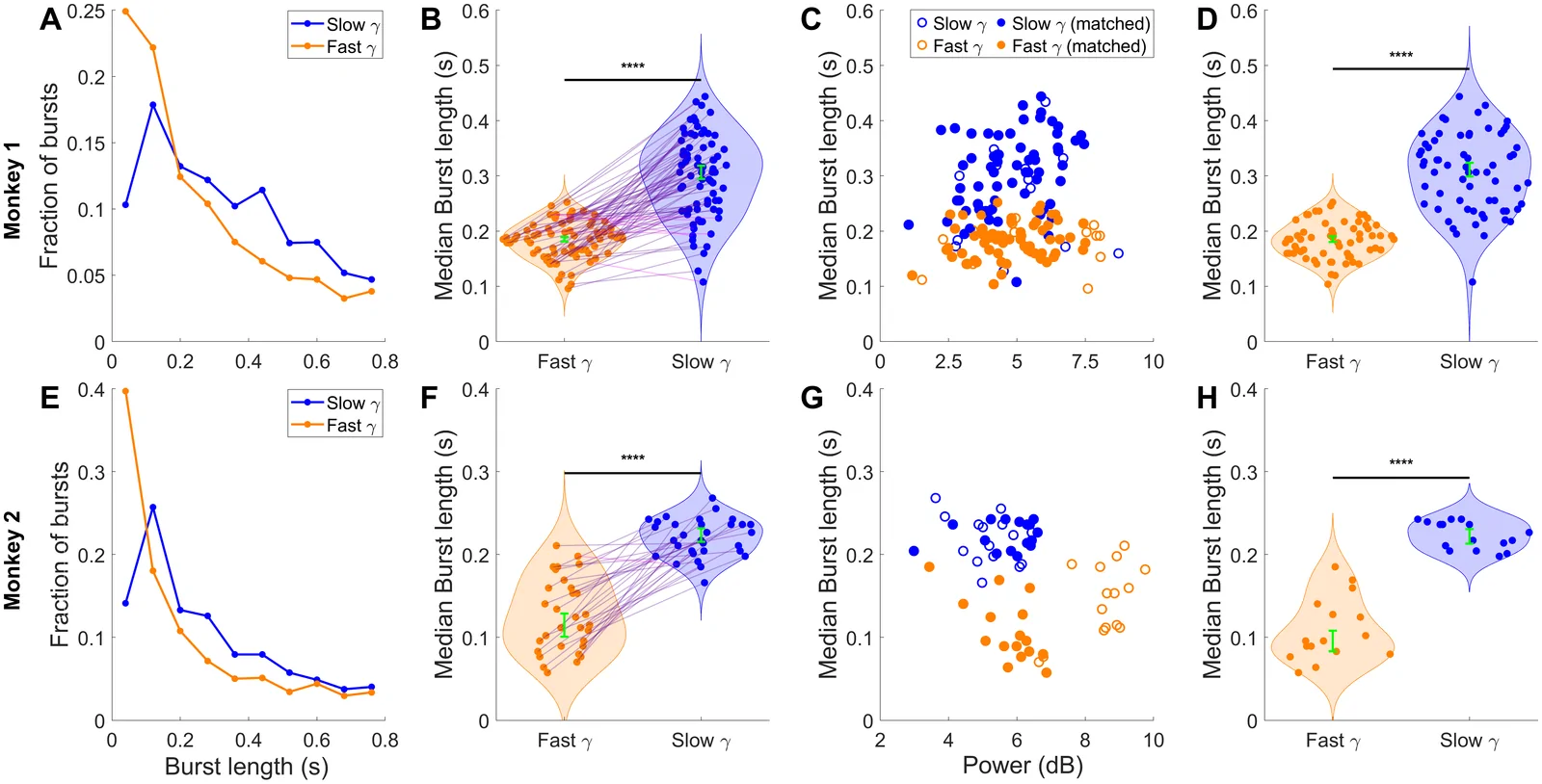
Comparison of burst durations of slow and fast gamma estimated by MP. (A, E) Histogram representing the distribution of burst durations of slow (blue) and fast (orange) gamma rhythm, combined across all electrodes of Monkey 1 and Monkey 2, when a stimulus with orientation of 157.5^o^ and spatial frequency 1 CPD was shown. (B, F) Median burst lengths (electrode-wise) of slow and fast gamma (*N* = 77 for Monkey 1 and *N* = 31 for Monkey 2, where N denotes the number of electrodes considered for analysis). The green error bar represents the overall median along with the standard error of the median. (C, G) Median burst lengths of both rhythms plotted versus normalized power (dB) for all the sites of Monkey 1 and Monkey 2. Filled circles represent the set of randomly selected power-matched electrodes having similar power in slow and fast gamma. (D, H) Median burst lengths of slow and fast gamma for the power-matched electrodes (*N* = 64 for Monkey 1 and *N* = 16 for Monkey 2 after power matching). The green error bar represents the overall median along with the standard error of the median.

Notably, there was a more pronounced heavy right-tail or right-shifted peak observed in the case of slow gamma, indicating that the proportion of longer burst durations observed was greater in the case of slow gamma than fast gamma. The burst length distributions of the two rhythms were significantly different (KS-test; *p*-value equals 9.81 x 10^-80^ and 1.39 x 10^-109^ for Monkey 1 and Monkey 2, respectively). Further, slow gamma rhythm exhibited significantly longer median burst lengths than fast gamma for both monkeys (Fig. 2B, F: Wilcoxon signed-rank test; *N* = 77, *p* = 4.88 x 10^-14^, W-stat = 2968, Z-value = 7.44 for Monkey 1 and *N* = 31, *p* = 7.42 x 10^-7^, W-stat = 494, Z-value = 4.81 for Monkey 2).

In our previous studies, we have observed a weak positive correlation between gamma burst durations and power in some cases, which was also observed for slow gamma in Monkey 1 (Spearman, r = 0.28, *p* = 1.35 x 10^-2^; Fig. 2C; for other conditions, the correlations were not significant). To rule out the potential bias due to power differences between the rhythms, we considered a subset of electrodes having similar slow and fast gamma power (see Methods section for details; the selected electrodes are indicated by solid circles in Fig. 2C and G). Even after power matching, slow gamma rhythm exhibited significantly longer median burst durations than fast gamma. (Fig. 2D, H: Wilcoxon rank-sum test; *N* = 64, *p* = 9.07 x 10^-19^, W-stat = 5968, Z-value = 8.77 for Monkey 1 and *N* = 16, *p* = 7.52 x 10^-7^, W-stat = 392, Z-value = 4.81 for Monkey 2). The trend was consistent across all stimulus orientations, despite distinct power tuning curves exhibited by slow and fast gamma rhythms as mentioned above (Supplementary Fig. S4 and Table S1).

### 3.4 Analysis of onset time and center frequencies of bursts

Here, we analyzed the temporal profiles of the slow and fast gamma bursts. Onset time for a given trial was defined as the time point at which the first (earliest) burst started (i.e., burst_center – burst_length/2). The estimated mean onset times of the slow gamma bursts were significantly higher than those of fast gamma (Fig. 3A, B, D, E; Two-sample paired t-test; *N* = 77, *p* = 1.39 x 10^-7^, t-stat = 5.64 for Monkey 1 and *N* = 31, *p* = 2.15 x 10^-10^, t-stat = 9.06 for Monkey 2). Thus, we observed an increased latency for the onset of slow gamma bursts (Supplementary Fig. S4 and Table S2).

**Fig. 3.**
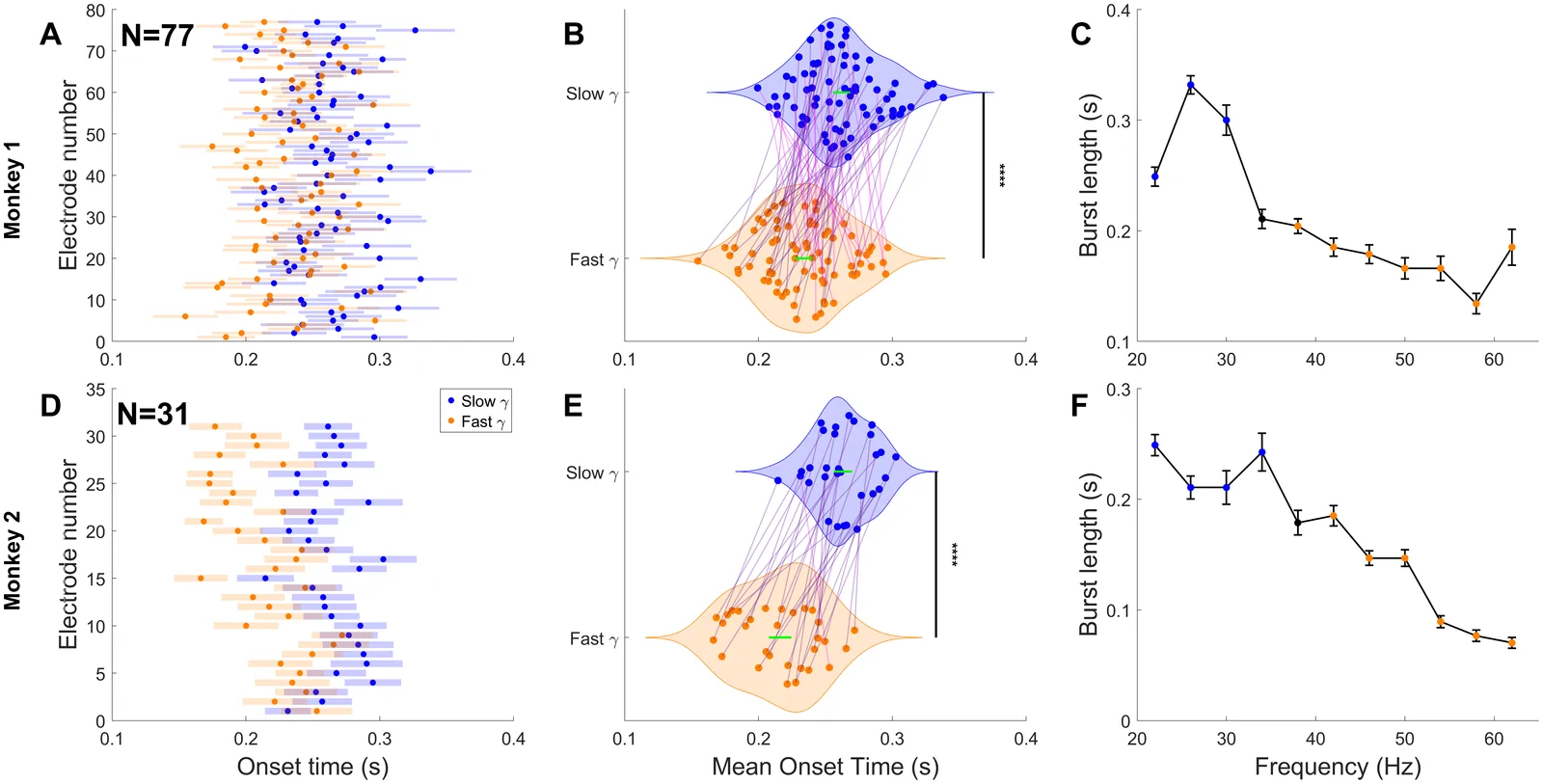
Analysis of the onset time and center frequencies of detected bursts. (A, D) Mean and standard error of the mean (SEM) of estimated onset times of the earliest observed burst in each trial for slow (blue) and fast (orange) gamma rhythm of Monkey 1 (*N* = 77) and Monkey 2 (*N* = 31) for stimulus orientation of 157.5^o^. (B, E) Mean onset times (electrode-wise) of slow and fast gamma bursts (*N* = 77 for Monkey 1 and *N* = 31 for Monkey 2). The green error bar represents the overall mean along with the SEM. (C, F) Estimated burst lengths are plotted versus their frequency center of the bursts (bin size equals 4 Hz). Error bars represent the median and standard error of the median of detected burst lengths combined across all sites. Frequency bin centers in slow and fast gamma frequency ranges are represented by blue and orange dots, respectively.

We also tested the burst durations as a function of the center frequency of the burst. Bursts were consistently longer in the range corresponding to slow gamma rhythm than fast gamma. There was no major inflation or irregularity observed in burst lengths at the power line frequency (50 Hz), as our recordings were conducted in a heavily shielded Faraday cage, which aided in attenuation of line noise (Fig. 3C, F). Similar results were observed for other methods as well, including HT and OMP-GEAR (Supplementary Fig. S5).

### 3.5 Analysis of number of cycles, burst PSD, occupancy, and overlap

Since slow gamma has lower frequency but larger duration than fast gamma, comparison in terms of the number of cycles instead of absolute time could potentially reveal interesting circuit properties. There were no significant differences between the median number of cycles of slow and fast gamma bursts for both monkeys (Supplementary Fig. S6A, C; Wilcoxon signed-rank test; *N* = 77, *p* = 0.11 for Monkey 1 and *N* = 31, *p* = 0.43 for Monkey 2). However, the distribution of the number of cycles differed between the two rhythms, with fast gamma bursts exhibiting a greater proportion of higher number of cycles (Supplementary Fig. S6B, D; KS-test; *p*-value equals 4.05 x 10^-59^ and 1.62 x 10^-33^ for Monkey 1 and Monkey 2, respectively). A smaller number of burst cycles were more common for both slow and fast gamma, but the number of cycles did not exceed ~30 cycles for slow gamma (which is understandable, as the total duration could not exceed 800 ms). For fast gamma, the fraction of longer bursts (>600 ms) was only slightly smaller than slow gamma (Fig. 2A and E), leading to a higher fraction of large burst cycles (Supplementary Fig. S6B, D).

We also tested for the presence of bimodality in the burst center frequencies by plotting the fraction of bursts detected as a function of their center frequency (Supplementary Fig. S7A, E), which revealed two peaks, weakly resembling the shape of PSD plots for both the monkeys (Supplementary Fig. S1A, C). To test for any relationship between slow and fast gamma bursts, we computed the spectrum at the time center of the bursts by first generating a two-dimensional time–frequency spectrum using MP and subsequently taking the one-dimensional vector of power values as a function of frequency centered at each slow and fast gamma burst and subsequently averaging them to get a single “burst PSD” each of slow and fast gamma (Supplementary Fig. S7B, F). We also shuffled the time centers randomly to generate the pseudo spectra of the two rhythms. While the power was higher at the respective burst frequencies by construction (i.e., a bump in slow gamma range for the slow gamma burst PSD), the power of the slow gamma burst PSD was comparable with the shuffled PSD in the fast gamma range (same for the fast gamma burst PSD that was overlapping with the shuffled PSD in the slow gamma range). This suggests that slow- and fast gamma burst time centers do not typically co-occur (which would have produced a bump in both bands). Further, this suggests that the detected fast gamma bursts were not false positives due to the harmonics of the slow gamma rhythm, as there was no elevation in fast gamma band for the slow gamma burst PSD compared with the shuffled PSD and vice versa.

To quantify how often these bursts occur and how much they overlap, we first computed the burst occupancy, which is the total time duration (out of 0.8 seconds) when a burst was present in a trial (Supplementary Fig. S7C, G). The mean burst occupancy was high (~0.49 seconds for Monkey 1 and ~0.46 seconds for Monkey 2) but did not differ between slow and fast gamma rhythms for the set of power-matched electrodes (Supplementary Fig. S7C, G; Two-sample unpaired t-test; *N* = 64, *p* = 0.44 for Monkey 1 and *N* = 16, *p* = 0.91 for Monkey 2, respectively). As expected from the high burst occupancy durations, there was a non-zero overlap between slow and fast gamma bursts in most of the trials (83.9% and 89.9% trials for Monkey 1 and Monkey 2, respectively), with an overall mean overlap duration of around 0.30 and 0.29 seconds for the two monkeys. As a control, we also computed the cross-trial overlap durations between slow and fast gamma after shuffling the order of trials for each electrode. However, the mean overlap duration in the actual dataset was not significantly different when compared with the trial-shuffled control (Supplementary Fig. S7D, H; Two-sample paired t-test; *N* = 77, *p* = 0.28 for Monkey 1 and *N* = 31, *p* = 0.55 for Monkey 2). Therefore, despite some overlap in burst durations, there was no evidence of coupling or interaction between slow and fast gamma bursts.

### 3.6 Functional connectivity in slow and fast gamma bands

We studied the functional connectivity of slow and fast gamma by computing the phase locking value (PLV) and weighted phase lag index (WPLI) profiles as a function of frequency, for trials corresponding to all the stimulus orientations, and electrode pairs were grouped according to the interelectrode distance (Fig. 4A, C, E, G). While PLV is a measure of phase consistency across trials between two signals, irrespective of the difference in phase angle, WPLI is typically used in EEG studies to reduce the effect of volume conduction because it measures the strength of non-synchronous (i.e., phase difference between signals that is different from zero) coupling (Vinck et al., 2011). Together, PLV and WPLI can be used to measure the strength of phase coupling between signals.

**Fig. 4.**
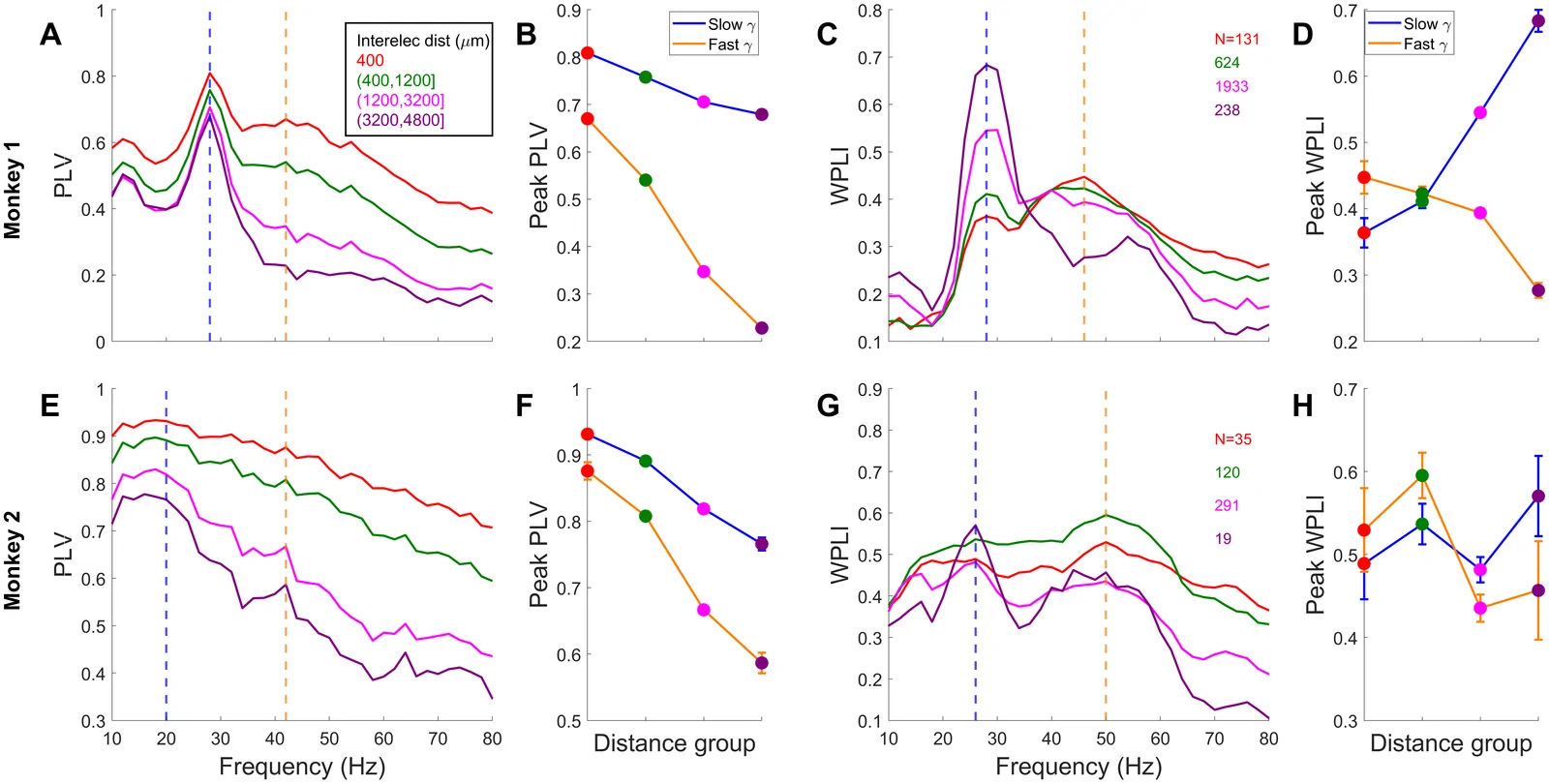
PLV and WPLI profiles of slow and fast gamma rhythm. (A, E) Mean phase locking value (PLV) or phase coherence plotted against frequency, with electrode pairs grouped according to their interelectrode distance (in µm). Colored legends indicate the range of interelectrode distances considered for the corresponding group. The number of electrode pairs falling in the respective groups for Monkey 1 (N_Total_ = ^77^C_2_ = 2926) and Monkey 2 (N_Total_ = ^31^C_2_ = 465) is reported in (C, G), respectively (for all stimulus orientations combined). The blue dotted line indicates the frequency in the range of slow gamma corresponding to Peak PLV. The orange dotted line indicates the same in the fast gamma frequency range. (B, F) Average Peak-PLV values (taken at the indicated frequency) are plotted against the respective groups, with increasing interelectrode distances. The colored error bar represents the overall mean along with the standard error of the mean. (C, G) Same as (A, E), for the weighted phase lag index (WPLI) method. (D, H) Same as (B, F), for Peak WPLI estimates.

PLV remained high in the slow gamma range as the interelectrode distance increased, while it decayed more rapidly for fast gamma, as shown previously (Fig. 4B, F; Murty et al., 2018; Two-way ANOVA with PLV estimates in the gamma bands and interelectrode distances as factors resulted in a highly significant interaction; *F*_(3,5851)_ = 537.60, *p* = 1.52 x 10^-308^ for Monkey 1 and *F*_(3,929)_ = 21.12, *p* = 3.08 x 10^-13^ for Monkey 2). Interestingly, for slow gamma, WPLI values were much smaller than PLV at small inter-electrode distances and either increased (Monkey 1) or remained the same (Monkey 2) with increasing inter-electrode distances (Fig. 4D, H; Two-way ANOVA with WPLI estimates in the gamma bands and interelectrode distances as factors resulted in a significant interaction; *F*_(3,5851)_ = 98.71, *p* = 2.53 x 10^-62^ for Monkey 1 and *F*_(3,929)_ = 2.72, *p* = 0.04 for Monkey 2). It is unclear why WPLI increased with inter-electrode distances for Monkey 1. One possibility is that since this measure indexes coupling strength with non-zero phase lag, synchronous coupling (i.e., occurring with phase lag of zero) is penalized. Thus, if slow gamma coupling had a stronger contribution from synchronous activity (zero phase difference) than from non-synchronous activity at small inter-electrode distances, WPLI would be lower and subsequently increase with increasing inter-electrode distance. Unlike slow gamma, fast gamma WPLI also decreased with increasing inter-electrode distance in both monkeys, suggesting more local origins of fast gamma.

### 3.7 JS model with noisy inputs reproduces the differential burst dynamics

Finally, we tested whether these burst characteristics can be replicated in a stochastic JS model with inputs modulated by OU noise. To generate gamma oscillations at different frequencies, we varied the firing rate time constant of inhibitory neuronal population to represent proxy SOM ($\tau_{I}=\tau_{SOM}=$ 15.4 ms) and PV ($\tau_{I}=\tau_{PV}=$ 7.7 ms) interneurons similar to previous studies (Methods section; Garcia Del Molino et al., 2017), which generated slow and fast gamma, respectively. Additionally, there were no recurrent connections among SOM interneurons ($W_{II}=0$ for the slow gamma model; Pfeffer et al., 2013). Figure 5A and E summarizes the model schematics used for simulating the two rhythms, with parameter values as mentioned in Tables 1, 2, and Methods section. Average raw time–frequency spectrograms and power spectral densities (Fig. 5B, C, G) demonstrate the generated slow and fast gamma rhythms in our modeling framework for a representative parametric combination. It verifies that $\tau_{I}$ determines the center frequency of the simulated oscillation, as known from the literature (Buzsáki & Wang, 2012).

**Fig. 5.**
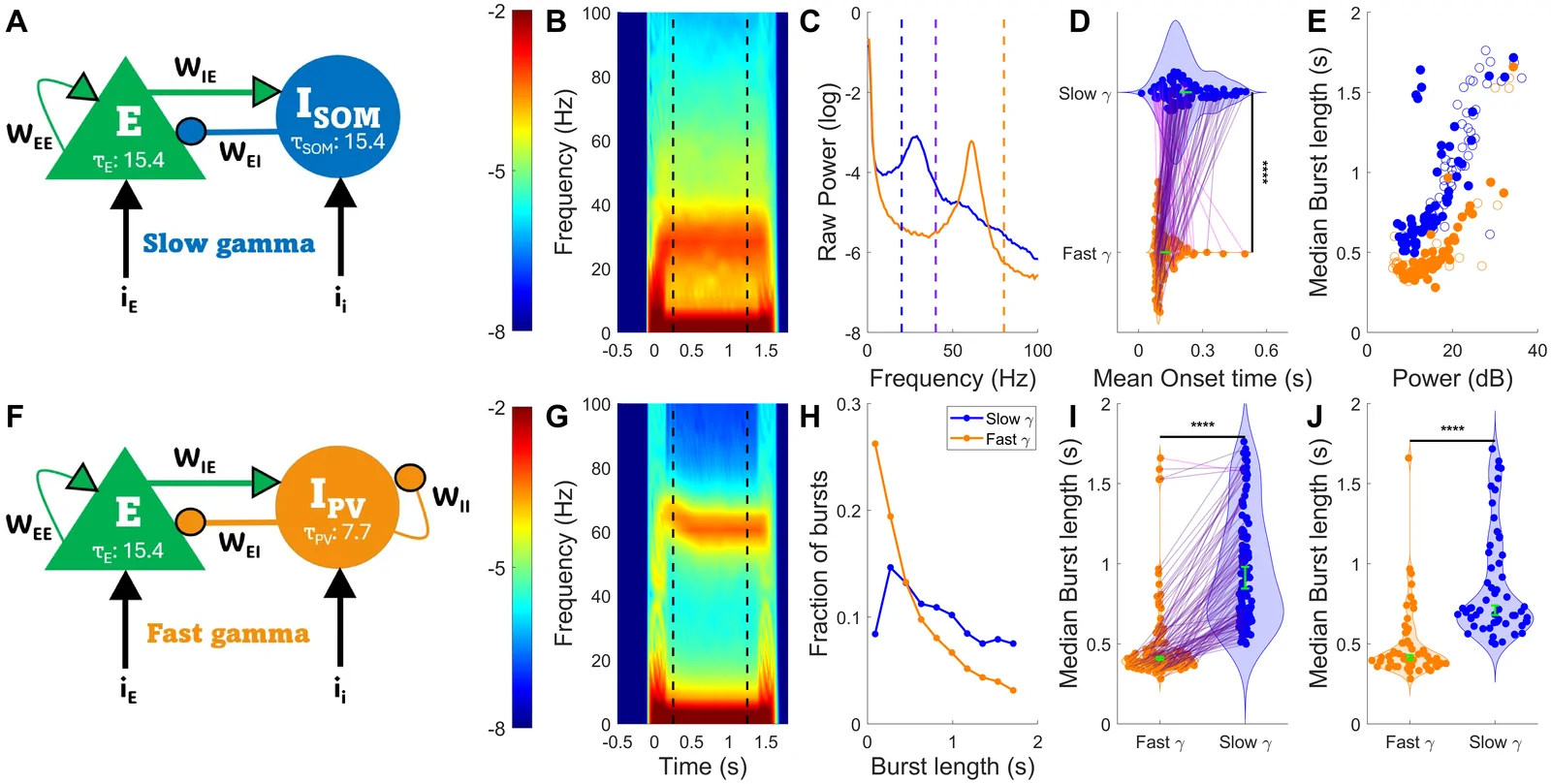
Analysis of simulated model outputs. (A, F) The model schematics are used for generating slow and fast gamma rhythms, respectively. Full set of parameters is reported in Tables 1 and 2. (E) Refers to excitatory population (pyramidal neurons), while SOM and PV represent somatostatin and parvalbumin-positive interneuronal populations (inhibitory), respectively. (B, G) Average raw time–frequency plots (over 50 iterations) for simulated proxy LFP from the two models for given representative parametric combinations, confirming the generation of slow and fast gamma, respectively. Colorbar indicates the raw power on a logarithmic scale. Vertical black dotted lines represent the stimulus period (0.25–1.25 seconds). (C) Average raw power spectral density plots (log scale) from the two models (blue: slow gamma, orange: fast gamma) are shown for the same parameters given as (B, G). (H) Histogram representing the distribution of burst durations of slow (blue) and fast (orange) gamma rhythm estimated by MP across all parametric conditions. (D) Mean onset times (across distinct parametric combinations (*N* = 105), with 50 iterations each) of slow and fast gamma bursts. The green error bar represents the overall mean along with the standard error of the mean. (I) Median burst lengths (across distinct parametric combinations (*N* = 105), with 50 iterations each) of slow and fast gamma. The green error bar represents the overall median along with the standard error of the median. (E) Median burst lengths of both rhythms plotted versus normalized power (dB) for all the specified parametric combinations. Filled circles represent the set of randomly selected power-matched conditions, having similar power of slow and fast gamma. (H) Median burst lengths of slow and fast gamma are represented for the power-matched conditions (*N* = 59). The green error bar represents the overall median along with the standard error of the median.

Subsequently, we performed similar MP analysis on the simulated proxy LFP. We used 128 different parametric combinations, spanning a wide range of mean input drives and OU noise parameters, in order to effectively vary the power and spectral variance of the generated rhythms (see Methods section). Interestingly, simply changing the inhibitory time constant was sufficient to replicate other characteristics of the bursts. First, a more prominent heavy tail was observed for slow gamma as compared with fast gamma rhythm in the burst duration distributions of the two rhythms, which were significantly different (Fig. 5H; KS-test; *p*-value less than 10^-308^, combined across all parametric combinations). Second, slow gamma exhibited longer median burst lengths and delayed onset than fast gamma in the modeling analysis across the different combinations. Bursts of both slow and fast gamma rhythms were detected in 105 parametric combinations (Fig. 5D, I; Two-sample paired t-test for mean onset time; *N* = 105, *p* = 2.36 x 10^-11^, t-stat = 7.35; Wilcoxon signed-rank test for median burst length; *N* = 105, *p* = 3.41 x 10^-19^, W-stat = 5560, Z-value = 8.88). As in real data, median burst lengths were higher for slow gamma even after performing power matching. (The selected conditions are indicated by solid circles in Figure 5E and J: Wilcoxon rank-sum test; *N* = 59, *p* = 1.26 x 10^-13^, W-stat = 4870.5, Z-value = 7.32.)

Differences in the inhibitory time constant across monkeys could also potentially explain differences in other properties. For example, the slow gamma was slightly faster in Monkey 2 than in Monkey 1. If the difference was due to a faster time constant in Monkey 2, burst lengths would also be shorter in this monkey, which was indeed the case (Fig. 2, B versus F). However, these correlations need to be interpreted with caution. For example, parameters of OU noise ($\theta_{E}$, $\theta_{I}$) also influence the burst durations and center frequencies of the rhythm by dictating the temporal dynamics of the input in our framework (Krishnakumaran & Ray, 2023). We observed that the absolute median burst length as well as peak frequencies of both slow gamma and fast gamma generally decreased with either increasing $\theta_{E\;}$ or $\theta_{I}\;$ (Supplementary Fig. S8) as shown previously (Krishnakumaran & Ray, 2023). Thus, if the differences in slow gamma peak frequency between the two monkeys were due to differences in $\theta_{E\;}$ or $\theta_{I}$, we would have expected longer burst durations for Monkey 2, opposite to what we found. Importantly, when compared at an identical set of parameters of noisy inputs, median duration of slow gamma remained longer than fast gamma.

## 4 Discussion

We compared the burst statistics of stimulus-induced slow and fast gamma oscillations in the primary visual cortex of non-human primates using several burst detection algorithms. We observed longer bouts and increased latency of stimulus-induced slow gamma rhythm as compared with fast gamma. The two rhythms had differential PLV and WPLI profiles, with higher long-range synchronization in slow gamma range than in fast gamma. Surprisingly, simply increasing the inhibitory firing rate time constant in a WC model operating in an ISN range was sufficient to not only produce slower rhythms, but also increase their duration and onset latency. Thus, simply slowing down the rhythm also made it longer and delayed its onset.

### 4.1 Gamma bursts in rodents and primates and importance of burst statistics

Rodent V1 shows multiple gamma rhythms, including an extremely narrow-band gamma around ~60 Hz that is enhanced by locomotion, arousal, and light intensity, suppressed by visual stimulation, and has thalamic or even retinal origins (Niell & Stryker, 2010; Saleem et al., 2017; Storchi et al., 2017). In addition, presentation of gratings induces oscillations in the slow gamma (20–30 Hz; also referred to as beta in several studies) and fast gamma (65–80 Hz) ranges, which have been linked to somatostatin (SOM) and parvalbumin-positive (PV+) interneurons, respectively (Chen et al., 2017; Veit et al., 2017). Broadband activation has also been reported in the gamma (20–80 Hz) and in the high gamma or epsilon range (80–150 Hz) as well (Meyerolbersleben et al., 2025). Such distinct gamma bursts are associated with complex visual features in naturalistic stimuli and are also modulated by the behavioral context (Meyerolbersleben et al., 2025; Perrenoud et al., 2025).

In cat (where stimulus-induced narrowband gamma was first reported; Gray et al., 1989) or primate V1, narrowband gamma is induced by presentation of specific visual stimuli such as bars or gratings, and the induced gamma is highly dependent on the properties of the stimulus such as its size, orientation, contrast, spatial frequency, and temporal frequency (see Murty et al., 2018, for references). In particular, the fast gamma in cats or primates appears to be very different from the fast gamma around ~60 Hz in rodents, since the latter has thalamic or retinal origins and is suppressed by visual stimulation.

It is unclear why only certain visual stimuli produce such robust narrowband gamma. One possibility is that it depends on the implementation of the spatio-temporal receptive field (RF) structure of the neurons. In primate V1, neurons have orientation tuned RFs with an excitatory center and inhibitory flanks (Hubel & Wiesel, 1968), which are, therefore, highly selective for oriented bars or gratings. Thus, appropriately tuned oriented bars or gratings are likely to engage local excitation–inhibition mechanisms. In V1, where features such as orientation, spatial frequency, and ocular dominance are tiled efficiently (Nauhaus et al., 2016), stimuli such as gratings are likely to produce large-scale synchronized excitation–inhibition mechanisms that produce salient gamma oscillations.

The physiological gamma rhythm burst statistics were shown to be generated in a specific optimum parametric space based on a stochastic neural network, with variations in network connectivity parameters leading to pathological burst statistics (Powanwe & Longtin, 2019). A few other theoretical studies have emphasized the role of transient gamma bursts in inter-areal communication across the cortex (Lowet et al., 2017; Palmigiano et al., 2017). Therefore, the observed differential burst properties of slow and fast gamma suggest that the two rhythms have distinct mechanisms of generation. These two gamma oscillations have been shown to exhibit traveling waves in Macaque V1 (Gautham & Ray, 2024), which propagate in different uncorrelated directions. The longer lifetime of traveling waves produced by slow gamma, in comparison with fast gamma, is in accordance with our findings. We also observed distinct power-tuning curves for the two rhythms across stimulus orientations and spatial frequency, which is consistent with previous studies (Han, Wang, Yang, et al., 2021; Murty et al., 2018). We recently found that while fast gamma phase is coupled to power in frequencies above 150 Hz (which in turn is tightly coupled with multiunit firing around the microelectrode; Ray & Maunsell, 2011), slow gamma shows phase–amplitude coupling to frequencies between 80 and 150 Hz (Prabhu & Ray, 2024).

The inferences from our estimated PLV and WPLI profiles also indicate that fast and slow gamma rhythms reflect coupling at distinct spatial scales, that is, local and global, respectively. The field–field coherence measurements have been shown to be substantially higher for slow gamma than for fast gamma in studies in both mice and macaque V1, even for widely separated electrodes (Murty et al., 2018; Veit et al., 2017). Slow gamma was shown to be generated by long-range horizontal connections in a modeling study (Han, Wang, Wu, et al., 2021). These observations support the hypothesis that slow gamma serves as a global modulatory signal (Prabhu & Ray, 2024; Veit et al., 2023). Longer bouts of slow gamma lead to an increased number of oscillatory cycles per burst, which could potentially compensate for its lower frequency (Zheng et al., 2016) and engage large populations of neurons across the neocortex for extended durations. On the contrary, shorter transient bursts of fast gamma (typical gamma oscillation) could be optimum for local synchronization and influencing spikes, as indicated by a modeling study (Saraf & Young, 2021).

In a network in which short duration (a few cycles) gamma bursts are generated due to excitation–inhibition interactions (as implemented in our noisy network model) and whose properties depend on the input parameters (such as the excitatory or inhibitory inputs, time constant of inhibition, or the filtering characteristics of OU noise in our model), any cortical process that involves changing any of these parameters or other parameters such as synaptic weights would also lead to incidental changes in these rhythms (Doelling & Assaneo, 2021; Ray, 2022).

### 4.2 Biophysical mechanisms

Experimental studies in mouse V1 employing optogenetics have tried to decipher the specific mechanisms behind the generation of these distinct gamma rhythms (Chen et al., 2017; Veit et al., 2017). The perisomatic inhibition by parvalbumin-positive (PV+) interneurons has been shown to contribute to the production of fast gamma. However, the generation of slow gamma rhythm is mediated by the dendritic inhibition provided by somatostatin (SOM) interneurons, which leads to considerably slower negative feedback (E-I network), as compared with that provided by direct perisomatic inhibition by PV+ neurons (which acts as a shunt, directly inhibiting spike production). Furthermore, a study in the mouse hippocampus has shown that the kinetics of inhibitory post-synaptic currents (IPSC) mediated by GABA_A_ receptors is slower (latency in opening and closing) in the dendrites than in the soma of pyramidal cells (Huang et al., 2023). Similar variability in the properties of post-synaptic receptors along the somato-dendritic axis might be present in pyramidal cells of V1 as well. Therefore, these could be the plausible biophysical mechanisms behind the increased latency and longer durations of slow gamma bursts. However, despite slow gamma rhythm exhibiting a considerably higher proportion of longer burst lengths, the mode of the distribution still lies around 100 ms. We suspect the heterogeneity in the distribution of slow gamma burst durations could be linked to the rich repertoire of active dendritic conductances, which in turn exhibit variability across space and plasticity with time (Sinha & Narayanan, 2022) or to other possible sources of generation. VIP interneurons have been shown to aid in the elevated global coherence in slow gamma range by the modulation of SOM neurons in mice V1 (Veit et al., 2023).

Moreover, there are multiple diverse subtypes of SOM interneurons in the cortex, broadly including non-Martinotti cells (which target layer 4), along with the typical majority dendrite-targeting Martinotti cells (Naka et al., 2019; Wu et al., 2023). Both these subtypes of SOM exhibit delayed firing relative to stimulus onset and within the gamma cycle, but it was the non-Martinotti cell type that was strongly phase locked to slow gamma in the visual cortex of mice (Onorato et al., 2025). On the contrary, PV interneurons have been shown to be phase locked to both slow and fast gamma (Onorato et al., 2025; Veit et al., 2017). However, as mentioned earlier, there are key species-specific differences in slow and fast gamma rhythms between monkeys and rodents, and hence analogous optogenetic experiments and laminar recordings in macaques might help unravel the precise mechanisms underlying the generation of both gamma rhythms (El-Shamayleh & Horwitz, 2019; Steinmetz et al., 2021).

### 4.3 Modeling studies

Recently, an in-silico study recruiting a morphologically realistic 3D model of a pyramidal neuron in V1 has demonstrated that the dendritic rhythmic inhibition (specifically at slow gamma frequencies) efficiently modulates the dendritic Ca^2+^ and NMDA spikes (dendritic Na^+^ spikes were unaffected; Headley et al., 2024). Examining the putative reciprocal influence of dendritic currents on the generation of slow gamma in a modeling framework could be insightful, although it would require intensive computational resources. An alternative strategy is to employ simplified phenomenological network models, as done in this study. We were able to replicate the differential burst dynamics of slow and fast gamma in a stochastic JS model framework. Bursts manifested as noise-interrupted limit cycles in our model, and thus primarily a larger value of firing-rate time constant ($\tau_{I})$ defined for SOM as compared with PV inhibitory interneurons, lead to reduced susceptibility to noisy inputs, subsequently generating sustained longer bursts of slow gamma rhythm. Longer latency to the onset of the slow gamma bursts also followed trivially from the larger $\tau_{I\;}$ , since it took longer for the system to move to the regime where it produced salient gamma oscillations when the time constant was large. While the absolute value of median burst durations was influenced by the given parameters of the OU noise-mediated inputs (Krishnakumaran & Ray, 2023), the trend between the two rhythms remained consistent irrespective of parametric conditions. Thus, the $\tau_{I\;}$ values assigned to SOM and PV interneuron were similar to those used in a previous study (Garcia Del Molino et al., 2017) and determined the center frequency (Buzsáki & Wang, 2012) of the generated oscillation as well as the temporal evolution of its bursts in our model.

We kept the two inhibitory populations non-interacting, since our main goal was to test whether the burst characteristics could be explained by simply changing a time constant in the model. Previous studies have implemented network models with multiple inhibitory neuronal populations (two or three), such as a stabilized supralinear network regime in case of firing-rate models or employing spiking neural networks to propose specific functions of these inhibitory interneurons in mediating circuit dynamics (Bos et al., 2025; Holt et al., 2024; Ter Wal & Tiesinga, 2021; Veit et al., 2023). It could be possible to suitably modify the WC-type models to generate multiple gamma rhythms concurrently, manifesting as bursts and thereby validate our results in another framework. However, due to the asymmetric inhibition of PV by the SOM interneurons in V1 (Pfeffer et al., 2013) and incorporating the effects of modulatory inputs, competing slow and fast gamma rhythms could arise in such three-population network models that interact with each other in non-trivial ways (Keeley et al., 2017). Such models could explain how multiple classes of interneurons could synergistically modify spiking activity in a network.

### 4.4 Burst profiles as potential diagnostics

The causal influence of gamma rhythm on cognitive processes and well-being remains a matter of debate, as the existing evidence is mostly correlational. However, the potential role of these burst profiles as biomarkers for the diagnosis of cognitive deficits could not be ruled out. For example, burst properties of the beta rhythm have been shown to change significantly in subjects with Parkinson’s disease as compared with healthy controls (Vinding et al., 2020). Previous studies have extensively focused on the decline of trial-averaged power of gamma oscillations computed using conventional spectral analysis in subjects with neurological disorders (An et al., 2018; Murty et al., 2021), neglecting the bursty nature of gamma rhythm. Moreover, stimulus-induced slow gamma rhythm appears to be quite salient in Human EEG recordings, potentially due to its global nature (Murty et al., 2018).

Recent studies on electrocorticogram (ECoG) recorded from V1 of non-human primates have shown that its spatial spread remains relatively local (Dubey & Ray, 2019). Further, studies on ECoG signals recorded from human V1 have shown that gamma oscillations exhibit similar tuning with respect to stimulus features such as contrast, orientation (for gratings), and color (isoluminant hues) to that observed in monkeys (Bartoli et al., 2019, 2020; Li et al., 2022). Therefore, analyzing the burst statistics of slow gamma rhythm in Human ECoG, EEG, or MEG recordings and examining whether they change with the onset of cognitive impairments could be an intriguing question for the future.

## Supplementary Material

Supplementary Material

## Data Availability

The codes and data used for our entire analysis are available on the following GitHub repository: https://github.com/R-Vignesh410/Slow_Fast_Gamma_Bursts.git.
