# Supplementary material for: Differential burst dynamics of slow and fast gamma rhythms in macaque primary visual cortex
Source: Imaging Neurosci (Camb). 2026 Oct 22;4:IMAG.a.1404. doi: 10.1162/IMAG.a.1404 (PMC13697248; doi:10.1162/IMAG.a.1404)
Supplement: Supplementary file 1 [file IMAG.a.1404_supp.pdf]

Supplementary Material

Supplementary Discussion

In most burst detection methods, the power of a signal in a particular frequency band is estimated using a spectral estimator, and time points where this power exceeds some threshold are found to identify bursts (see (Chandran KS et al., 2018) for a detailed comparison of various methods). The main issue with this approach is that direct spectral estimators are themselves noisy and yield power values that are distributed exponentially irrespective of biological variability (see Figure 1 of (Chandran KS et al., 2018)), such that any rhythm appears bursty with a few large power values interspersed among many small values (as shown in Figure S3C). This issue is observed even when using Hilbert transform, in which band-pass filtering followed by the amplitude/power estimation step is similar to spectral estimation. MP-based methods solve this issue by decomposing the signal in the time domain itself using a large, overcomplete dictionary, such that spectral estimation is not needed at successive time points.

We note that different burst estimation methods are compared by generating synthetic signals in which synthetic gamma bursts of known durations are generated using Gabor atoms and injected into spontaneous LFP data. However, real gamma bursts often exhibit nonstationary frequencies (e.g., chirps), asymmetric rise and decay profiles, or distortions due to sharp transients and other noise sources, which are not captured in our simulations. Since MP and OMP-GEAR also decompose signals using Gabor atoms, this simulation framework is therefore particularly well matched to the MP-based approaches. However, we have previously observed that even when other spectral estimation-based methods employ Gabor basis functions, such as continuous

Gabor Transform or Morlet wavelets, the estimated bursts remain inaccurate (Figure 3 of (Chandran KS et al., 2018)). Further, other burst-detection methods often employ a filtering operation with a fixed band, which also cannot accurately decompose signals such as chirps or sharp transients (one exception is the Hilbert-Huang Transform, which uses empirical mode decomposition that can potentially address some of these issues (Huang et al., 1998)). Here, we test the performance of MP, OMP-GEAR and Hilbert transform using only Gabor gamma bursts; how different burst algorithms perform when the bursts are more complex remains an open question that requires future investigation. For a discussion on possible strategies, see (Anand et al., 2025).

Figure S2A shows the average power spectral density (PSD; averaged over 280 stimulus repeats combined across all eight stimulus orientations) for an example electrode of Monkey 1 for which both slow and fast gamma rhythms were prominently induced, as evident by two distinct 'bumps' in the PSD (Figure S2A; magenta trace). We injected Gabor atoms of pre-determined fixed durations (0.05 to 0.4 ms, with 0.05 ms step size) in the spontaneous LFP (green trace), with center frequencies randomly selected from a uniform distribution within the broad frequency range of 20-65 Hz, thus mimicking either the physiological slow or fast gamma rhythms. These bursts were then suitably scaled to ensure that the average PSD of the synthetic LFP signal matched that of the real data in the frequency range of our interest (Figure S2A). More details about synthetic LFP generation can be found in our previous studies (Anand et al., 2025; Chandran KS et al., 2018). Consistent with previous studies, MP and OMP-GEAR could robustly detect the injected bursts of varying durations, while the HT failed to efficiently compute burst lengths, especially for longer durations of bursts

injected (Figure S2B, root mean square error (RMSE) value of fitting: 0.0071, 0.0058, 0.1920 for MP, OMP-GEAR and HT, respectively).

How accurately do all methods identify bursts of a particular duration? Figure S2C shows the distribution of detected burst durations for the three methods when bursts of 0.3 s were injected. As shown previously (see Figure 4 of (Chandran KS et al., 2018)), while HT captures many spurious short bursts (<100 ms) due to the inherent variability in estimated values of power, MP and OMP-GEAR can better tolerate minor fluctuations in power or phase of the signals in burst detection, resulting in the mode of the distribution near the “true” burst length. Further, MP is able to reliably estimate the true burst length irrespective of its center frequency (Figure S2D). We computed the F1 score (see Methods) to compare the performance of all methods and found that MP and OMP-GEAR exhibited a better F-1 score than HT across injected burst lengths. (Figure S2E).

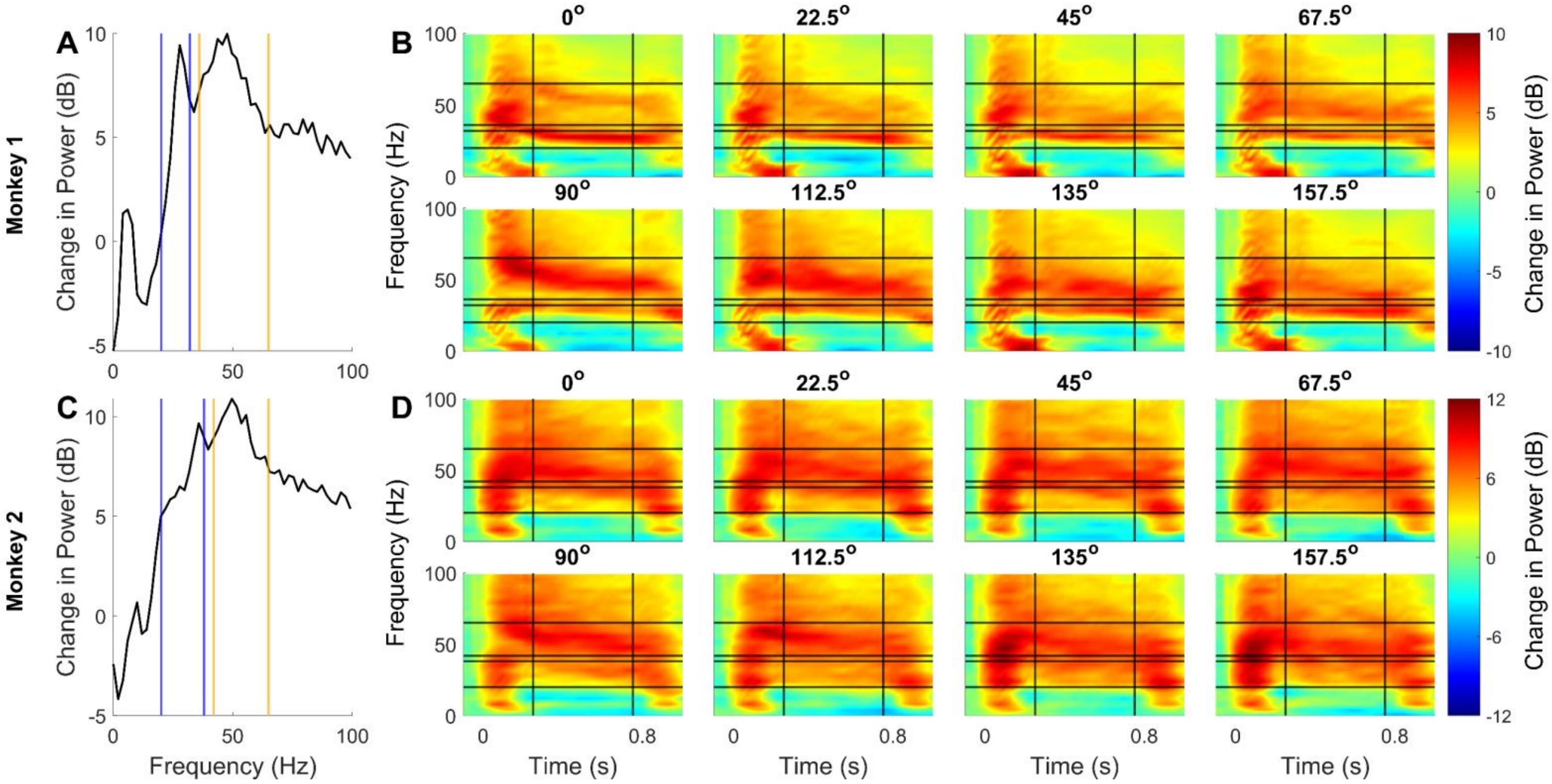

Figure S1: Representative average PSD traces and time-frequency spectrograms.

(A) Average normalized power-spectral density (with respect to baseline, dB scale) of LFP recordings from an example electrode in Monkey 1, for 280 trials corresponding to stimuli of all orientations and spatial frequency of 1 CPD. **(C)** same as **(A)**, for Monkey 2. The blue and orange solid lines represent the considered frequency ranges of slow and fast gamma, respectively, for the corresponding monkeys. **(B)** Time-frequency spectrogram (normalized with respect to baseline, colorbar denotes the power (dB scale)) estimated by multitaper method (single taper), for each of the eight orientations averaged across all the electrodes for Monkey 1. **(D)** same as **(B)**, for Monkey 2.

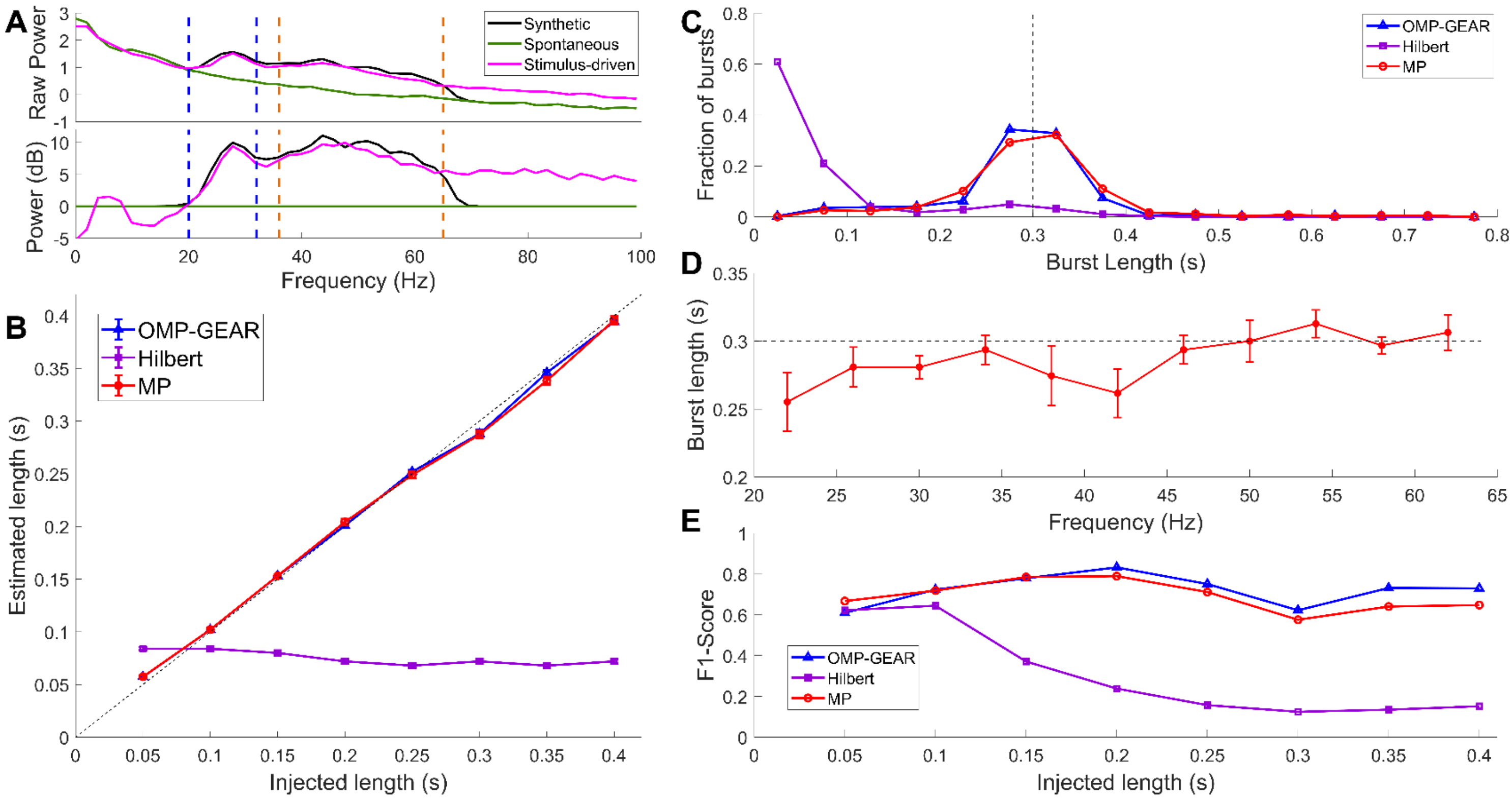

Figure S2: Performance of burst detection algorithms on synthetic LFP.

(A) Upper panel: Average power-spectral densities (PSD; log(µV^2/Hz)) of three signals: Stimulus-driven (magenta trace), Spontaneous (green trace), and Synthetic LFP (black trace). Stimulus-driven signal corresponds to real LFP recordings from the same example electrode in Monkey 1 as used in Figure S1, for which full-screen, full-contrast stimuli were shown for 280 trials (combined across all orientations). PSD was computed between 250 to 750 ms after stimulus onset. The PSD for spontaneous LFP was obtained between -500 to 0 ms of stimulus onset. Synthetic LFP refers to the signal constructed by injecting bursts of fixed duration into each trial of spontaneous LFP (see Methods for details). The blue and orange dotted lines represent the frequency ranges of slow (20-32 Hz) and fast gamma (36-65 Hz), respectively for Monkey 1. Lower panel shows the change in power between stimulus and baseline (converted to 'dB' scale). **(B)** Performance of the three methods: MP (red), OMP-

GEAR (blue), and HT (purple) on the synthetic LFP. The known burst lengths (50 ms to 400 ms, with 50 ms step size injected for 280 trials each) are plotted versus the median burst length along with the standard error of the median (SEM), as estimated by the three methods. The black dotted line denotes the 'y=x' line. **(C)** Histogram representing the distribution of detected burst lengths when bursts of 0.3 s were injected (represented by dotted black line). **(D)** Median burst durations along with the standard error of the median estimated by MP, as a function of their center frequency. The "true" burst length of 0.3 s is denoted by the black dotted line. **(E)** F1-scores (burst detection) for all the three methods as a function of injected burst duration.

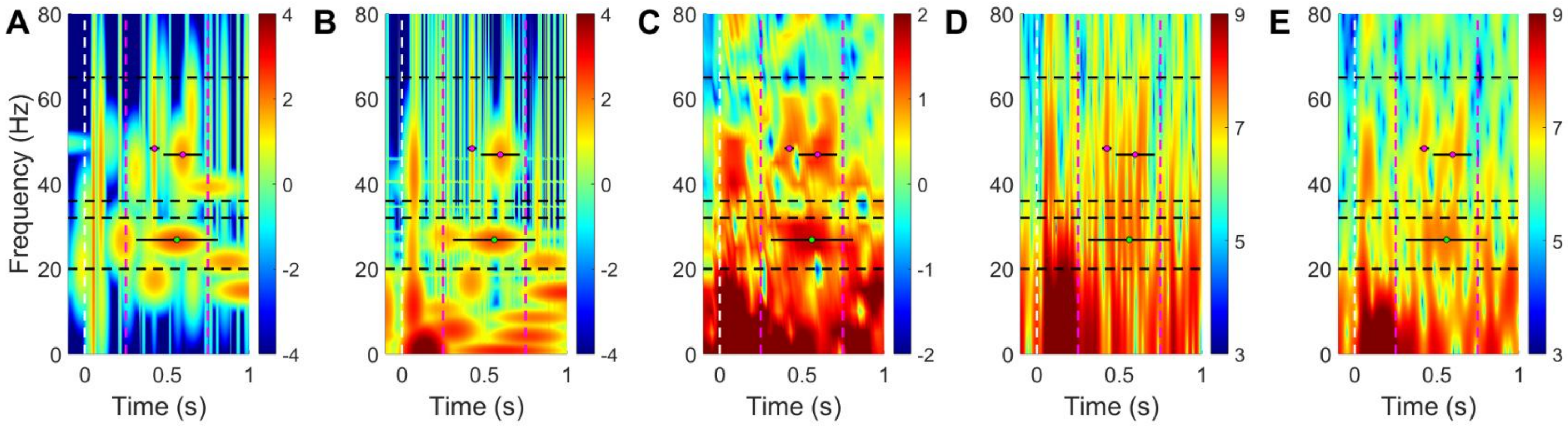

Figure S3: Time-frequency (TF) spectrograms estimated by different methods.

(A) The raw TF spectrum of an example single trial (with the signal filtered between 15-80 Hz) estimated by MP. **(B)** The raw TF spectrum of the same trial (unfiltered signal) constructed using MP algorithm with a higher number of iterations (200). **(C)** Same as **(B),** estimated using multitaper method (single taper; window of length 200 ms and step size of 20 ms). **(D)** Same as **(B),** constructed using the Continuous Gabor transform (CGT) with a window width of 12.5 ms. **(E)** Same as **(B),** but constructed using CGT with a window width of 25 ms. The black line segments in **(A-E)** represent the burst durations detected by MP for this single trial. Vertical magenta dotted lines represent the stimulus period (0.25 to 0.75 s), while the vertical white dotted line indicates the stimulus onset (0 s). Horizontal black dotted lines represent the frequency ranges of slow (20-32 Hz) and fast gamma (36-65 Hz) for Monkey 1. Colorbar indicates the estimated raw power on a logarithmic scale.

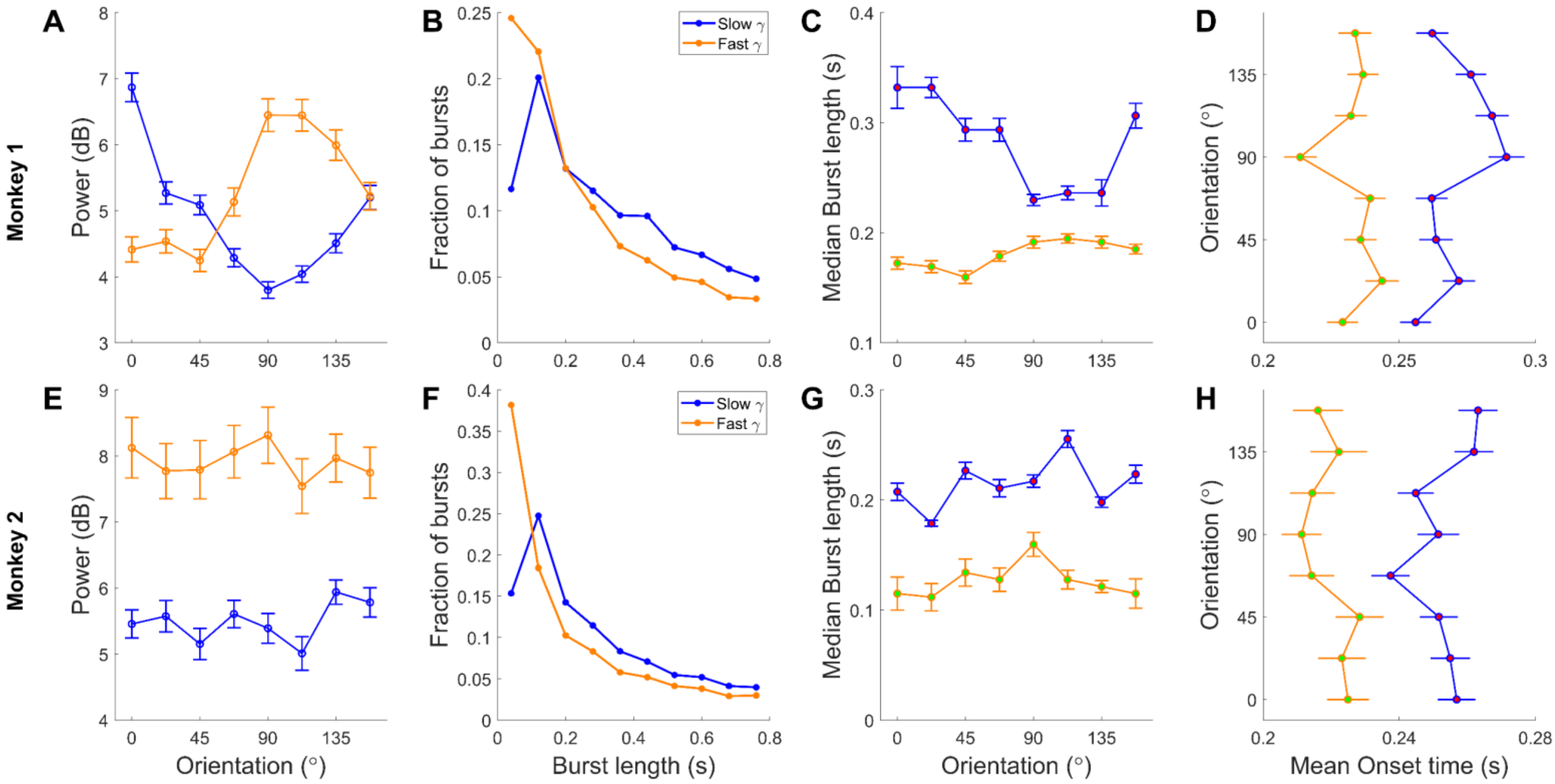

Figure S4: Power tuning, burst durations and onset times across all stimulus orientations.

(A, E) Normalized power tuning curves (dB) across different stimulus orientations of Monkey 1 and Monkey 2. Error bars indicate the mean and SEM (standard error of the mean) of power values across electrodes. **(B, F)** Histogram representing the distribution of burst durations of slow (blue) and fast (orange) gamma rhythm, combined across all electrodes and orientations of Monkey 1 and Monkey 2. A significantly higher fraction of longer bursts is observed for the slow gamma as compared to fast gamma rhythm (KS-test; p-value less than 10^{-308}). **(C, G)** Median and standard error of median of median burst lengths (electrode-wise) of slow and fast gamma for each orientation for Monkey 1 (N=77) and Monkey 2 (N=31). Median slow gamma burst durations were significantly longer than those of fast gamma at all orientations (Wilcoxon sign-rank; corresponding statistics are reported in Table S1).

(D, H) Mean and standard error of the mean (SEM) of the estimated mean onset times of slow (blue) and fast (orange) gamma rhythm for each orientation for Monkey 1 (N=77) and Monkey 2 (N=31). Mean onset times of slow gamma bursts were significantly delayed as compared to those of fast gamma at all orientations (paired t-test; corresponding statistics are reported in Table S2).

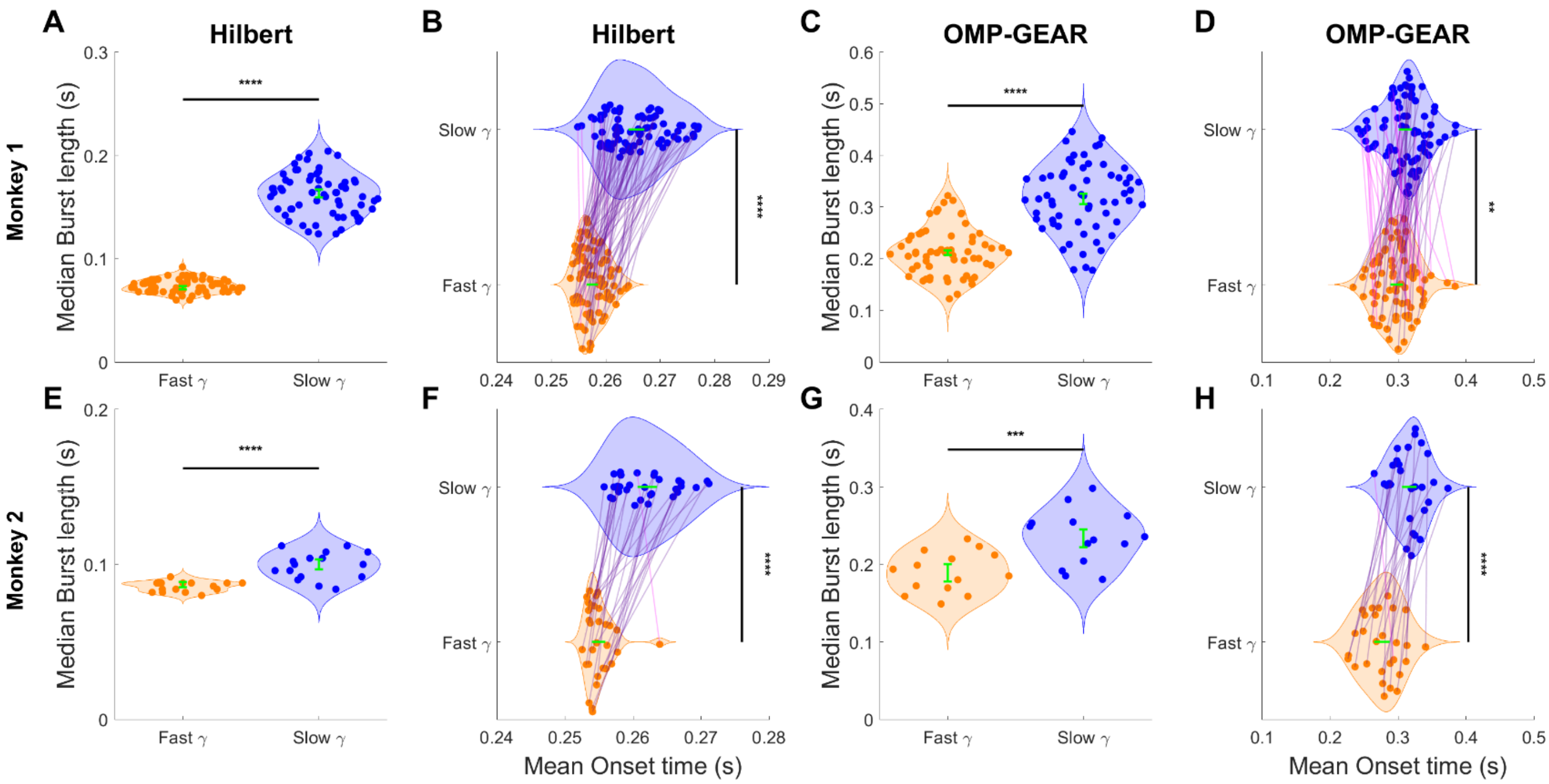

Figure S5: Results using HT and OMP-GEAR.

(A, E) Median burst lengths of slow and fast gamma computed by HT for the power-matched electrodes (Wilcoxon rank-sum test; N=64, p= 8.22 x 10^{-23}, W-stat= 6176, Z-value= 9.76 for Monkey 1 and N=16, p= 3.09 x 10^{-5}, W-stat= 370.5, Z-value= 4.01 for Monkey 2; for stimulus orientation equal to 157.5°). The green error bar represents the overall median along with the standard error of the median. **(C, G)** same as **(A, E),** for the OMP-GEAR method (Wilcoxon rank-sum test; N=61, p= 3.04 x 10^{-14}, W-stat= 5218, Z-value= 7.51 for Monkey 1 and N=14, p= 9.63 x 10^{-4}, W-stat= 271, Z-value= 3.10 for Monkey 2). **(B, F)** Mean onset times (electrode-wise) of slow and fast gamma bursts computed by HT for all electrodes (Two-sample paired t-test; N=77, p= 3.73 x 10^{-21}, t-stat= 12.91 for Monkey 1 and N=16, p= 4.51 x 10^{-10}, t-stat= 8.76 for Monkey 2). The green error bar represents the overall mean along with the standard error of the mean. **(D, H)** same as **(B, F),** for the OMP-GEAR method (Two-sampled paired t-

test; N=74, $p = 2.60 \times 10^{-3}$, t-stat= 2.88 for Monkey 1 and N=28, $p = 7.04 \times 10^{-8}$, t-stat= 7.05 for Monkey 2).

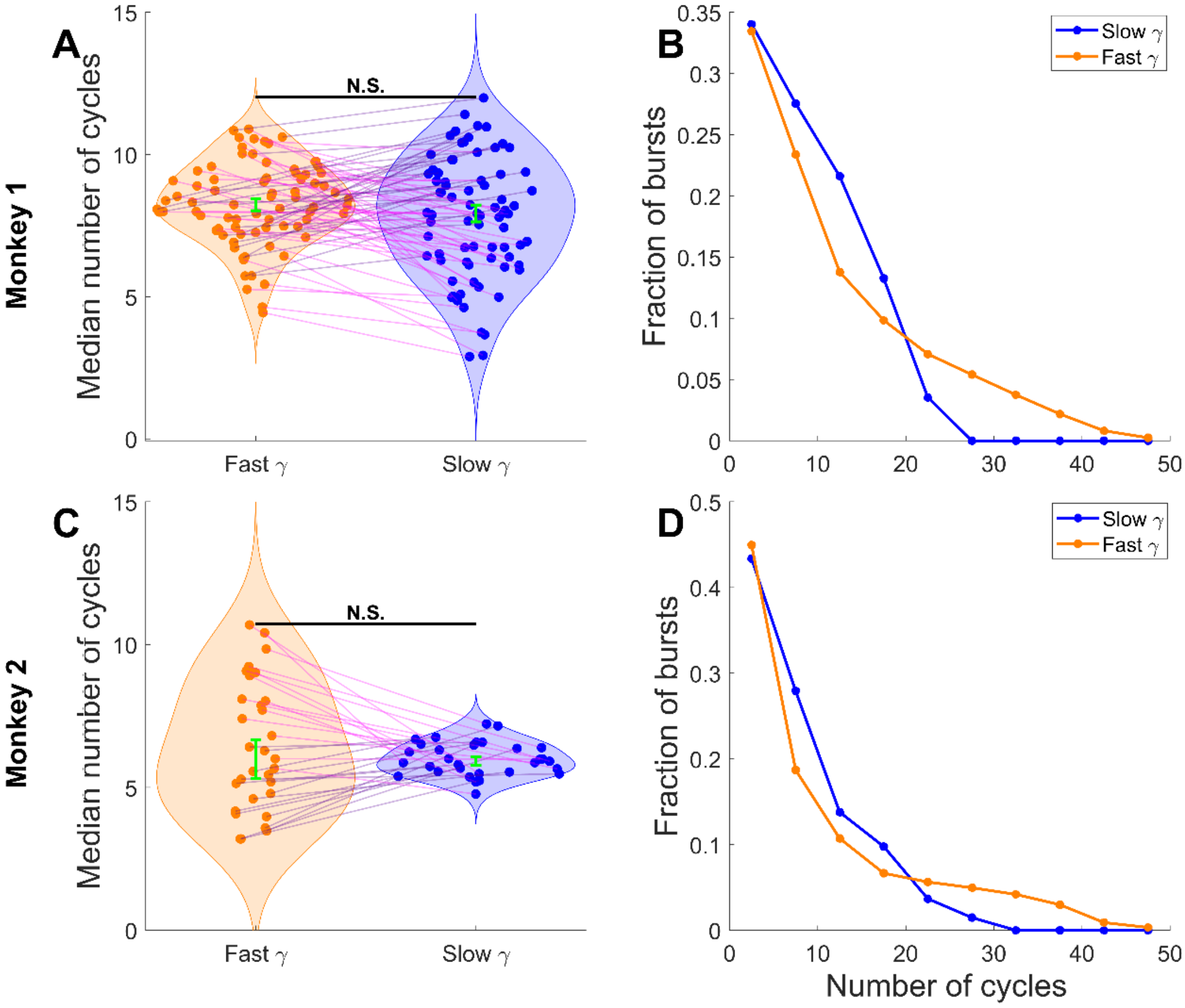

Figure S6: Analysis of the number of cycles

(A, C) Median number of cycles for slow and fast gamma (N=77 for Monkey 1 and N=31 for Monkey 2) for stimulus orientation equal to 157.5°. The green error bar represents the overall median along with the standard error of the median. **(B, D)** Histogram representing the distribution of number of cycles of slow (blue) and fast (orange) gamma rhythm, combined across all electrodes of Monkey 1 and Monkey 2.

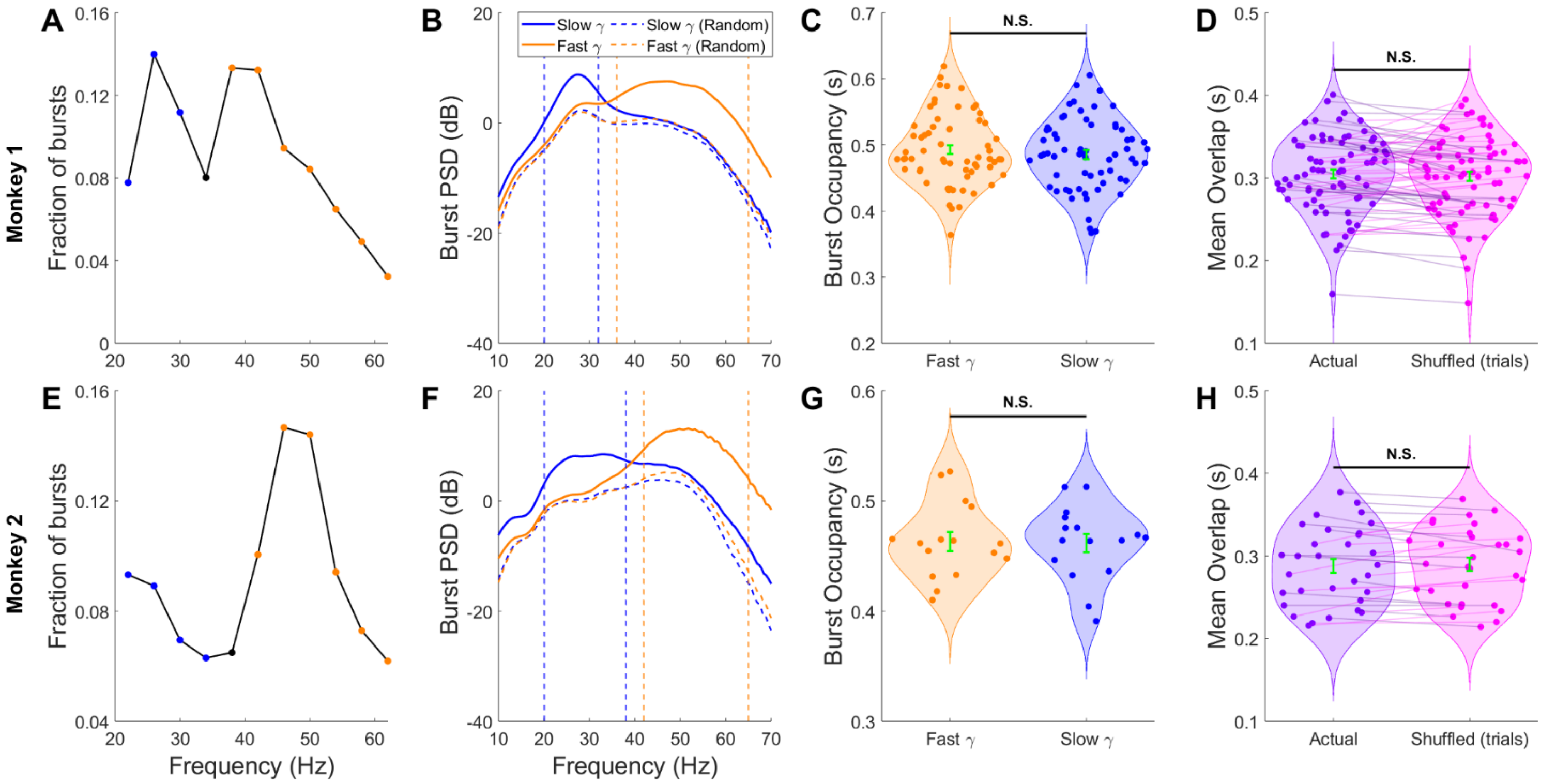

Figure S7: Distribution of burst-center frequencies, Burst PSD, occupancy, and overlap

(A, E) Fraction of detected bursts plotted versus their center frequencies (bin size equals 4 Hz) for stimulus orientation equal to 157.5°. Frequency bin centers in slow and fast gamma frequency ranges are represented by blue and orange colored dots, respectively. **(B, F)** Average burst-PSD (units: dB), normalized with respect to baseline period, constructed from MP algorithm for slow (blue) and fast (orange) gamma burst time centers (see text for details). The dotted versions of PSD traces correspond to those constructed from shuffled time centers of slow (blue) and fast (orange) gamma bursts. The traces were smoothened using MATLAB function 'movmean' using a window width of ≈4 Hz. The blue and orange vertical dotted lines represent the considered frequency ranges of slow and fast gamma, respectively, for the corresponding monkeys. **(C, G)** Mean burst occupancy (electrode-wise) of slow and

fast gamma rhythms for the same set of power-matched electrodes shown in Figure 2 (N=64 for Monkey 1 and N=16 for Monkey 2). The green error bar represents the overall mean along with the SEM. **(D, H)** Mean burst overlap duration (electrode-wise) between slow and fast gamma rhythms for the actual dataset (purple) and control dataset (pink) with shuffled order of trials (N=77 for Monkey 1 and N=31 for Monkey 2). The green error bar represents the overall mean along with the SEM.

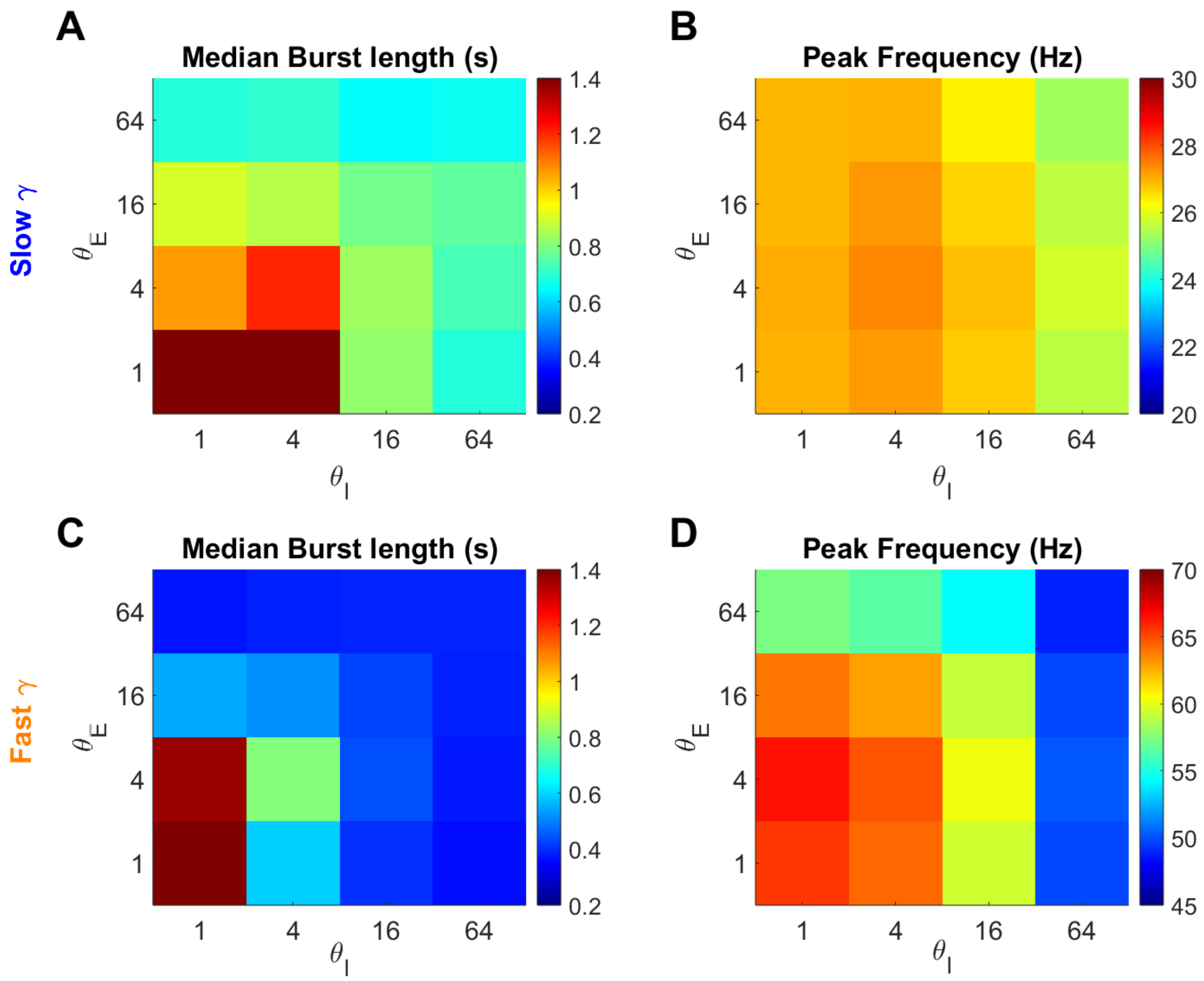

Figure S8: Effect of varying noise parameters (θ_E and θ_I) on burst duration and peak frequency.

(A) Median burst duration of slow gamma in seconds (colorbar) as a function of θ_E and θ_I (combined across all values of steady state input current). **(B)** Same as **(A),** but for mean peak frequency (Hz; colorbar) of slow gamma rhythm. **(C-D)** Same as **(A-B)**, but for fast gamma.

Table S1: Wilcoxon signed-rank test was performed for each of the orientations (median burst durations (electrode-wise)) as shown in Figure S4 C and G.

	Monkey-1 (N=77)			Monkey-2 (N=31)		
**Orientation (°)**	**p-value**	**Z-value**	**W-stat**	**p-value**	**Z-value**	**W-stat**
**0**	2.42×10^{-14}	7.54	2919	6.14×10^{-7}	4.85	496
**22.5**	1.39×10^{-13}	7.30	2940.5	1.42×10^{-6}	4.68	460.5
**45**	5.27×10^{-14}	7.43	2966	1.66×10^{-6}	4.65	459
**67.5**	2.68×10^{-13}	7.22	2923	1.66×10^{-6}	4.65	433
**90**	2.16×10^{-8}	5.48	2462.5	2.71×10^{-6}	4.55	480.5
**112.5**	3.84×10^{-7}	4.94	2418	8.67×10^{-7}	4.78	492.5
**135**	6.77×10^{-11}	6.42	2703.5	2.83×10^{-6}	4.54	480
**157.5**	4.88×10^{-14}	7.44	2968	7.42×10^{-7}	4.81	494

Table S2: Paired t-test was performed for each of the orientations (mean onset times (electrode-wise)) as shown in Figure S4 D and H.

	Monkey-1 (N=77)		Monkey-2 (N=31)	
**Orientation (°)**	**p-value**	**t-stat**	**p-value**	**t-stat**
**0**	4.60×10^{-9}	6.45	8.29×10^{-7}	5.94
**22.5**	9.91×10^{-7}	5.15	6.33×10^{-7}	6.03
**45**	4.96×10^{-8}	5.89	1.79×10^{-5}	4.85
**67.5**	6.98×10^{-8}	5.81	4.93×10^{-7}	6.12
**90**	3.28×10^{-22}	13.52	4.09×10^{-8}	7.03
**112.5**	3.77×10^{-17}	10.71	1.43×10^{-6}	5.74
**135**	2.48×10^{-13}	8.70	3.80×10^{-7}	6.22
**157.5**	1.39×10^{-7}	5.64	2.15×10^{-10}	9.06

References

Anand, Abhishek, Murthy, Chandra, & Ray, Supratim. (2025). Burst estimation through atomic decomposition (BEAD): A toolbox to find oscillatory bursts in brain signals. *Imaging Neuroscience*, *3*, IMAG.a.86. https://doi.org/10.1162/IMAG.a.86

Chandran KS, Subhash, Seelamantula, Chandra Sekhar, & Ray, Supratim. (2018). Duration analysis using matching pursuit algorithm reveals longer bouts of gamma rhythm. *Journal of Neurophysiology*, *119*(3), 808–821. https://doi.org/10.1152/jn.00154.2017

Huang, Norden E., Shen, Zheng, Long, Steven R., Wu, Manli C., Shih, Hsing H., Zheng, Quanan, Yen, Nai-Chyuan, Tung, Chi Chao, & Liu, Henry H. (1998). The empirical mode decomposition and the Hilbert spectrum for nonlinear and non-stationary time series analysis. *Proceedings of the Royal Society of London. Series A: Mathematical, Physical and Engineering Sciences*, *454*(1971), 903–995. https://doi.org/10.1098/rspa.1998.0193
